# Disruption of nucleocytoplasmic trafficking as a cellular senescence driver

**DOI:** 10.1038/s12276-021-00643-6

**Published:** 2021-06-29

**Authors:** Ji-Hwan Park, Sung Jin Ryu, Byung Ju Kim, Hyun-Ji Cho, Chi Hyun Park, Hyo Jei Claudia Choi, Eun-Jin Jang, Eun Jae Yang, Jeong-A Hwang, Seung-Hwa Woo, Jun Hyung Lee, Ji Hwan Park, Kyung-Mi Choi, Young-Yon Kwon, Cheol-Koo Lee, Joon Tae Park, Sung Chun Cho, Yun-Il Lee, Sung Bae Lee, Jeong A. Han, Kyung A Cho, Min-Sik Kim, Daehee Hwang, Young-Sam Lee, Sang Chul Park

**Affiliations:** 1 Korea Bioinformation Center, Korea Research Institute of Bioscience & Biotechnology, Daejeon, 34141 Republic of Korea; 2 Samsung Advanced Institute of Technology, Samsung Electronics Co., Ltd., Suwon, 16677 Republic of Korea; 3 Present Address: Well Aging Research Center, Division of Biotechnology, DGIST, Daegu, 42988 Republic of Korea; 4 Department of Computer Science and Engineering, Kangwon National University, Chuncheon, 24341 Republic of Korea; 5 Department of New Biology, DGIST, Daegu, 42988 Republic of Korea; 6 Department of Biotechnology, College of Life Sciences and Biotechnology, Korea University, Seoul, 02841 Republic of Korea; 7 Division of Life Sciences, College of Life Sciences and Bioengineering, Incheon National University, Incheon, 22012 Republic of Korea; 8 Department of Brain & Cognitive Science, DGIST, Daegu, 42988 Republic of Korea; 9 Department of Biochemistry and Molecular Biology, Kangwon National University School of Medicine, Chuncheon, 24341 Republic of Korea; 10 Department of Biochemistry, Chonnam National University, Medical School, Gwangju, 61469 Republic of Korea; 11 Department of Biological Sciences, Seoul National University, Seoul, 08826 Republic of Korea; 12 The Future Life & Society Research Center, Advanced Institute of Aging Science, Chonnam National University, Gwangju, 61469 Republic of Korea; 13 Present Address: UBLBio Corporation, Suwon, 16679 Republic of Korea

**Keywords:** Senescence, Gene ontology

## Abstract

Senescent cells exhibit a reduced response to intrinsic and extrinsic stimuli. This diminished reaction may be explained by the disrupted transmission of nuclear signals. However, this hypothesis requires more evidence before it can be accepted as a mechanism of cellular senescence. A proteomic analysis of the cytoplasmic and nuclear fractions obtained from young and senescent cells revealed disruption of nucleocytoplasmic trafficking (NCT) as an essential feature of replicative senescence (RS) at the global level. Blocking NCT either chemically or genetically induced the acquisition of an RS-like senescence phenotype, named nuclear barrier-induced senescence (NBIS). A transcriptome analysis revealed that, among various types of cellular senescence, NBIS exhibited a gene expression pattern most similar to that of RS. Core proteomic and transcriptomic patterns common to both RS and NBIS included upregulation of the endocytosis-lysosome network and downregulation of NCT in senescent cells, patterns also observed in an aging yeast model. These results imply coordinated aging-dependent reduction in the transmission of extrinsic signals to the nucleus and in the nucleus-to-cytoplasm supply of proteins/RNAs. We further showed that the aging-associated decrease in Sp1 transcription factor expression was critical for the downregulation of NCT. Our results suggest that NBIS is a modality of cellular senescence that may represent the nature of physiological aging in eukaryotes.

## Aging: Communication defects between nucleus and cytoplasm

Disruption of signals passing into or out of nucleus may be involved in aging. As cells age, they become senescent, i.e., stop growing and dividing. Cellular senescence is charateristic of reduced responsiveness to various stresses. Recent studies have noted that communication between nucleus and cytoplasm is dysfunctional in senescent cells. But it has not been determined whether the defects in this communication brings to cellular aging. Sang Chul Park and co-workers at DGIST, KRIBB, CNU and SNU in South Korea investigated nuclear–cytoplasmic trafficking (NCT) in senescent cells and found that impairment of NCT caused cells to become senescent. As cells aged, transmission of signals into or out of nucleus was reduced. Studying cells in which NCT is deliberately blocked may illuminate how cells age.

## Introduction

Cellular senescence is characterized by the arrest of cell proliferation, which can be induced by various intrinsic and extrinsic stress factors; according to these factors, cellular senescence can be categorized as replicative senescence (RS), which is caused by long-term cell division; oncogene-induced senescence (OIS); DNA damage-induced senescence (DDIS); and oxidative stress-induced senescence (OSIS). All these types of senescence share common senescent hallmarks, such as permanent growth arrest, genomic instability, senescence-associated β-galactosidase (SA-β-gal) expression, the senescence-associated secretory phenotype, and heterochromatin foci formation^1^ in addition to common transcriptomic signatures of cellular senescence^2^. However, several differential senescence-associated features unique to these individual modalities lead to the question of the nature of physiological aging. The shared and distinctive features of the individual cellular senescence models have been extensively reviewed^3–6^.

An increasing amount of evidence has indicated a novel senescence-associated feature, i.e., resistance to intrinsic and extrinsic signals^7–9^. This resistance can be explained by a novel concept, i.e., dysfunctional transmission of signals between the cytoplasm and nucleus^10–13^. Under normal conditions, nucleocytoplasmic trafficking (NCT) proteins^14^ select signals that can pass through the nuclear membrane through a process called selective permeability during signal transduction. For example, NCT is activated by the Ran protein gradient across a nuclear pore^13^. Disruption of the Ran gradient is frequently observed in senescent cells, including DDIS models^15^, and in fibroblasts of patients with Hutchinson-Gilford progeria syndrome^16^. Correspondingly, depletion of the Ran protein in mouse embryonic fibroblasts accelerates cellular senescence^17^. Aged yeast cells also show decreased transport across the nuclear membrane^18^. Moreover, the nuclear entry of Sp1, a key transcription factor that induces the expression of NCT-related genes, is decreased in senescent cells^19^. Furthermore, secondary mitotic and apoptotic signals, such as signals transmitted through the p-ERK, p-P38, and p-Jun kinase signaling pathways, are trapped in the cytoplasm of senescent cells^8,19–23^. Considering the findings showing that NCT is disrupted during cellular senescence, we previously proposed a nuclear barrier hypothesis of aging^12,24^. The nuclear barrier is a functional concept formed on the basis of dysregulated NCT proteins in senescent cells. This barrier disrupts the selective permeability of signals passing through the nuclear membrane, which is actively modulated by intact NCT proteins. This nuclear barrier hypothesis was originally based on the expression of several molecular markers; however, for the nuclear barrier formation and functional implications to be realized, systematic global proteome and transcriptome analyses are required.

In this study, we initially performed proteomic analysis of cytoplasmic and nuclear fractions from young and senescent cells to analyze aging-dependent nucleocytoplasmic protein segregation patterns. The expression of NCT and RNA processing-/transport-related proteins was downregulated in the nucleus of senescent cells, but the expression of endocytosis-lysosome pathway-related proteins was upregulated. In contrast, the expression of immune response-related proteins was downregulated in the cytoplasm. These aging-dependent differences in nucleocytoplasmic protein distribution support the presence of a nuclear barrier in senescent cells. Blocking NCT to induce a nuclear barrier led to the acquisition of a senescence phenotype referred to as nuclear barrier-induced senescence (NBIS), and changes in the nuclear and cytoplasmic proteomes were found to be similar to those associated with RS. A transcriptomic analysis revealed that NBIS was associated with senescence-dependent expression patterns that with most similar to those associated with RS than with other types of senescence (OIS, DDIS, or OSIS). Moreover, a nuclear barrier was also found in an aging yeast model. We further showed that age-associated downregulation of Sp1 transcription factor expression induced downregulation of NCT-related gene expression. Taken together, the results of our integrative analysis implicate NBIS as a novel mechanism of cellular senescence that is most similar to RS and demonstrate that nuclear barrier induction is an important characteristic of the physiological aging of eukaryotes.

## Results

### Proteomic analysis reveals the presence of a nuclear barrier in senescent cells

**Fig. 1:**
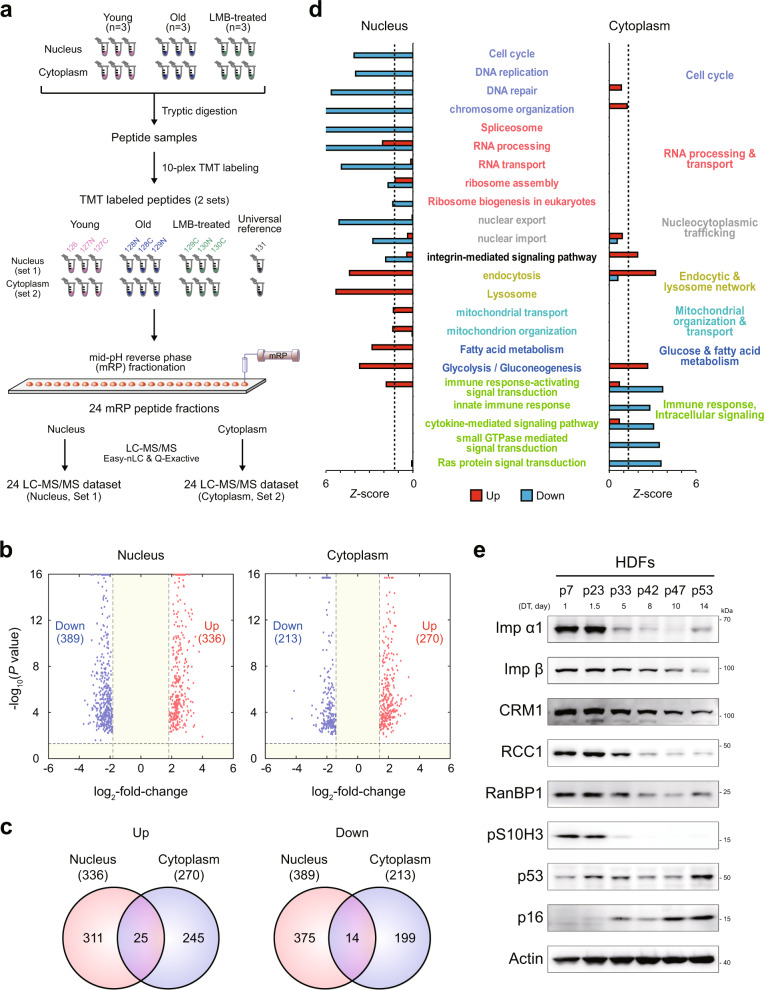
Proteomic analysis reveals the presence of a nuclear barrier in senescent cells. **a** Overall scheme of proteomic analysis of nuclear and cytoplasmic fractions of young, senescent (RS), and leptomycin B-treated (LMB-NBIS) HDFs. Three biological replicates (*n* = 3) were analyzed. Sets 1 and 2, nuclear and cytoplasmic fractions, respectively; tandem mass tag (TMT) 126, 127N, and 127C for young HDFs; TMT 128N, 128C, and 129N for RS; and TMT 129C, 130N, and 130C for LMB-NBIS. **b** Volcano plots showing upregulated (red) and downregulated (blue) proteins in the nucleus (left) and cytoplasm (right). **c** Relationships between upregulated (left) or downregulated (right) proteins in the nucleus and cytoplasm of RS. **d** Cellular processes (GOBPs) significantly (*P* < 0.1) enriched by upregulated (red) or downregulated (blue) genes in RS. The Z-score indicates –N^-1^(*P*), where *P* is the enrichment *P* value determined with DAVID software and N^−1^(*·*) is the inverse normal distribution. **e** Immunoblot of NCT-related proteins using whole-cell lysates of HDFs at different passages during senescence. Doubling times (DTs) corresponding to passage numbers are shown.

According to the nuclear barrier hypothesis, proteins are distributed separately in the cytoplasm and nucleus of senescent cells. To test this hypothesis, we isolated the cytoplasmic and nuclear fractions of young (control) and senescent cells (*n* = 3) with doubling times of 1 and 14 days, respectively, and then performed comparative proteomic analysis of these fractions. After tryptic digestion of each sample, we performed tandem mass tag (TMT) labeling on the digested peptides of the young and senescent cells in the nuclear fractions (Set 1) and on those of the young and senescent cells in the cytoplasmic fractions (Set 2) (Fig. 1a). The labeled peptide samples in Set 1 were fractionated into 24 fractions using mid-pH reverse-phase fractionation, and each fraction was then analyzed using liquid chromatography-tandem mass spectrometry analysis (LC-MS/MS); the same process was used to fractionate the peptide samples in Set 2 (Fig. 1a). An MS-GF+^25^ database search of the peptides obtained by LC-MS/MS led to the identification of 99474 unique peptides with a false discovery rate (FDR) criterion of 1% and 4967 protein groups with two or more unique sibling peptides (Supplementary Table 1).

To examine senescence-dependent changes in nucleocytoplasmic protein distribution, we compared the intensities of the TMT reporter ions and identified 8599 and 11254 differentially expressed peptides (DE peptides) in the cytoplasm and nucleus between young and senescent cells, respectively. We then identified 725 and 483 differentially expressed proteins (DEPs) in the nucleus (336 upregulated and 389 downregulated in senescent cells) and the cytoplasm (270 upregulated and 213 downregulated), respectively, with each DEP associated with two or more unique DE peptides that showed consistent up- or downregulation (Fig. 1b, c; Supplementary Table 2). Among these DEPs, 39 (25 upregulated and 14 downregulated) were common between the cytoplasmic and nuclear fractions. Consistent with our previous finding^10,19^, 32 NCT-related proteins were downregulated in the nucleus of senescent cells compared to that of young cells, including RNA-binding proteins (RANBP2/3), nucleoporins (NUP98/133/155), mRNA export system-related proteins (RCC1, DDX39B, SARNP, and THO complex proteins THOC2/5), and lamin A/C (LMNA).

For the systematic analysis of cellular processes affected by the aging-dependent differential nucleocytoplasmic protein distribution, we performed enrichment analysis of Gene Ontology biological processes (GOBPs) and Kyoto Encyclopedia of Genes and Genomes (KEGG) pathways for the up- and downregulated proteins in the cytoplasm or nucleus. The upregulated proteins in the nucleus of senescent cells were mainly associated with the endocytic and lysosome network (endocytosis and lysosomes), glucose and fatty acid metabolism, and mitochondrial organization and transport (Fig. 1d). The downregulated proteins in the nucleus were associated with the cell cycle (DNA replication), NCT (nuclear export and import), RNA processing and transport (spliceosome and ribosome assembly), and integrin-mediated signaling pathways. On the other hand, the downregulated proteins in the cytoplasm were associated with the innate immune response and intracellular signaling (cytokine-mediated signaling and regulation of Ras and small GTPase signaling) (Fig. 1d). The Western blot analysis of human diploid fibroblasts (HDFs) after different passages during RS confirmed that the expression of NCT-related proteins was downregulated (Fig. 1e).

Interestingly, the proteomic analysis revealed that a number of proteins showed opposite alteration patterns in the nucleus and cytoplasm. For example, the expression of 28 proteins was upregulated in the nucleus and downregulated in the cytoplasm, suggesting that they predominantly accumulate in the nucleus and are potentially associated with NBIS phenotypes. Western blotting confirmed the up- and downregulation of the expression of two representative proteins (ADRM1 and HSP90B1) in the nucleus and cytoplasm, respectively (Supplementary Fig. S1a). Moreover, we found that neither protein size, mass, isoelectric point (pI), nor disordered region accounted for the nuclear or cytoplasmic enrichment of these DEPs, suggesting that factors other than physiochemical and structural factors may contribute to protein trafficking in the presence of the nuclear barrier in senescing cells (Supplementary Fig. S1b). Taken together, these data suggest that downregulated NCT and RNA processing and transport form a nuclear barrier in senescent cells. This nuclear barrier may result in the accumulation of proteins involved in the endocytic pathway, metabolism, and mitochondrial organization in the nucleus, and nuclear trapping of the endocytic pathway then downregulates the immune response.

### A chemically induced nuclear barrier leads to RS-like senescence

**Fig. 2:**
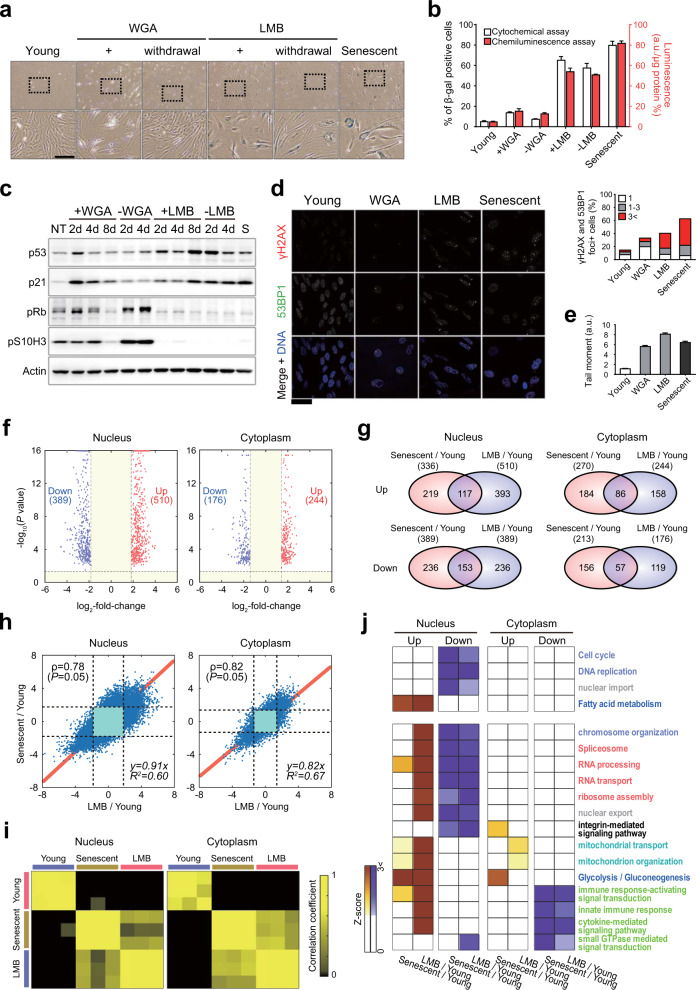
A chemically induced nuclear barrier leads to RS-like senescence. **a** HDFs stained for SA β-gal activity by the cytochemical (X-gal) method. Young HDFs were treated with 6 μg/ml wheat germ agglutinin (WGA) or 0.1 μg/ml leptomycin B (LMB) for 8 days and then cultured for an additional 4 days after WGA and LMB removal. Bottom panels show 3× magnified versions of images, as indicated by the dashed box on top. Scale bar, 50 μm. **b** Percentages of β-gal-positive cells (*n* > 200 cells). Left (white) and right (red) bars indicate the percentages estimated by cytochemical and chemiluminescence assays, respectively. The means ± SD (*n* = 3) are reported; a.u., arbitrary unit. **c** Immunoblot using whole-cell lysates of young HDFs (NT), young HDFs treated with WGA or LMB, or RS HDFs (S). **d** Immunocytochemical detection of DNA damage repair markers (53BP1 and γH2AX foci) in HDFs treated with WGA or LMB. Scale bar, 50 μm. Quantitative analysis of γH2AX and 53BP1 double-positive nuclear foci was performed using micrographs of cells labeled with DNA damage repair markers (*n* > 200 cells). **e** Comet assay of young HDFs, WGA- and LMB-treated young HDFs, and RS HDFs (*n* > 50 cells). **f** Volcano plots showing upregulated (red) and downregulated (blue) proteins in the nucleus (left) and cytoplasm (right). **g** Relationships between upregulated (top) or downregulated (bottom) proteins in the nucleus (left) and cytoplasm (right) of cells undergoing RS or LMB-NBIS. **h** Scatter plots showing significant (*P* < 0.05) Pearson correlations (ρ) of log_2_-fold-changes for the differentially expressed sibling peptides with consistent alteration directions for all DEPs measured from the nucleus (left) and cytoplasm (right) between cells undergoing RS and LMB-NBIS. Red lines denote regression lines, and the regression equations are shown together with *R*^2^ values. **i** Heat maps showing correlations of protein levels measured from individual samples of young, senescent (RS), and LMB-treated (LMB-NBIS) HDFs. Color bar, gradient of the Pearson correlation. **j** Heat map showing the gene enrichment patterns of GOBPs and KEGG pathways by upregulated and downregulated proteins in the nucleus or cytoplasm of cells undergoing RS or LMB-NBIS. Color bar, gradient of Z-score for the enrichment *P* value determined with DAVID software.

Despite the association found between the nuclear barrier and cellular senescence, it is still unclear whether the nuclear barrier is a cause or result of cellular senescence. Thus, we first examined the effect of the nuclear barrier on cellular senescence in human diploid fibroblasts (HDFs) by treating these cells with leptomycin B (LMB) and wheat germ agglutinin (WGA), which inhibit nuclear export and import, respectively. After treatment with LMB, the percentage of SA-β-gal-positive HDFs increased significantly to more than 60% compared with that in young cells (approximately 5%) (Fig. 2a, b). Interestingly, even when the HDFs were cultured continuously for 4 days after LMB withdrawal, the increase in SA-β-gal activity was not reversed, illustrating the irreversible nature of LMB-induced senescence. In contrast, after treatment with WGA, the percentage of SA-β-gal-positive HDFs increased to approximately 15% but was restored to normal levels after withdrawal of WGA (Fig. 2a, b), revealing the reversible nature of WGA-induced senescence.

Next, we examined the effects of LMB and WGA on the cell cycle by monitoring the levels of cell cycle markers. The levels of p53 and p21, which are G0/G1 checkpoint regulators, increased only in the LMB-treated cells, and the increases in these levels were maintained even after LMB withdrawal (Fig. 2c). In addition, LMB or WGA treatment decreased the levels of phosphorylated H3 Ser-10 (pS10H3), a mitosis marker, and phosphorylated Rb (pRb), a G_1_/S marker (after 8 days of WGA treatment and after 2 days with LMB treatment; Fig. 2c). The decreases in these levels were reversed after WGA withdrawal but not after LMB withdrawal. Next, we compared the effects of LMB and WGA on DNA damage by staining for γH2AX and p53 binding protein 1 (53BP1). The percentage of HDFs with γH2AX and 53BP1 double-positive nuclear foci, which indicated DNA double-strand breaks (DSBs), increased to approximately 35% of total cells after WGA treatment and to more than 40% after LMB treatment, compared with approximately 15% of young HDFs (Fig. 2d). Correspondingly, neutral comet assays showed that the number of DSBs increased after LMB and WGA treatment (Fig. 2e). Notably, LMB treatment led to a greater increase in the number of DSBs than WGA. Collectively, these data indicate that the inhibition of NCT by LMB treatment can result in strong irreversible NBIS.

We performed further proteomic analysis of the cytoplasmic and nuclear fractions of young HDFs treated with or without LMB (Fig. 1a). Only the effects of LMB on nuclear and cytoplasmic proteomes were analyzed because LMB induced a stronger irreversible senescence phenotype similar to RS than was induced by WGA. We then identified 899 DEPs (510 upregulated and 389 downregulated) and 420 DEPs (244 upregulated and 176 downregulated) in the nucleus and cytoplasm, respectively, of the LMB-treated cells (Fig. 2f). A comparison of these DEPs with those in senescent cells showed significant (*P* < 0.05) overlapping in both the cytoplasm and nucleus (Fig. 2g). Correspondingly, there were significant (*P* < 0.05) correlations between the changes in the expression of proteins between the LMB-treated cells and the young cells (LMB/Young) and senescent cells compared to young cells (Senescent/Young) (Fig. 2h and Supplementary Fig. S2a), and the proteome profiles of the cytoplasm and nucleus of the LMB-treated cells were more highly correlated with those of senescent cells than with those of young cells (Fig. 2i).

To examine cellular processes affected by LMB treatment, we performed GOBP and KEGG pathway enrichment analysis for the up- and downregulated proteins in the nucleus or cytoplasm of the LMB-treated cells. The analysis showed that fatty acid metabolism was upregulated in the nucleus, but the cell cycle, DNA replication, and NCT (nuclear import) were downregulated, consistent with the findings with senescent cells (Fig. 2j, top heat map). Splicing, RNA processing/transport, and ribosome assembly were consistently downregulated in the nuclei of senescent and LMB-treated cells (Fig. 2j). However, the proteins involved in these processes showed mixed up- and downregulation in the LMB-treated nuclei (Supplementary Fig. S2b–e). To clarify the mixed alteration patterns, we built a network model describing the interactions among the proteins involved in these processes. The network model shows that splicing is coordinately increased from pre-mRNA, 5′-end capping, and Complex A-C to intron and 3′-end processing with no U5 splicing, whereas mRNA transport, ribosome assembly, and formation of translation complexes are coordinately decreased (Supplementary Fig. S2f). Moreover, mitochondrial organization/transport and innate immune response/cytokine-mediated signaling were consistently upregulated in the nucleus and downregulated in the cytoplasm of senescent and LMB-treated cells, respectively; however, they also showed the opposite patterns in the other compartments with mixed up- and downregulation patterns, possibly due to the characteristics of young cells even in the presence of a nuclear barrier. Finally, we confirmed the predominant accumulation of the representative proteins in the nucleus (PSMB4, ADRM1, CTSD, and HEXB) or cytoplasm (HSPD1, CALD1, MYO6, and EPLIN) after LMB treatment (Supplementary Fig. S3). All these data suggest that cells undergoing NBIS, particularly those induced by nuclear export inhibition, show RS-like senescence phenotypes and changes in nucleocytoplasmic proteome distribution.

### A genetically induced nuclear barrier also results in RS-like senescence

**Fig. 3:**
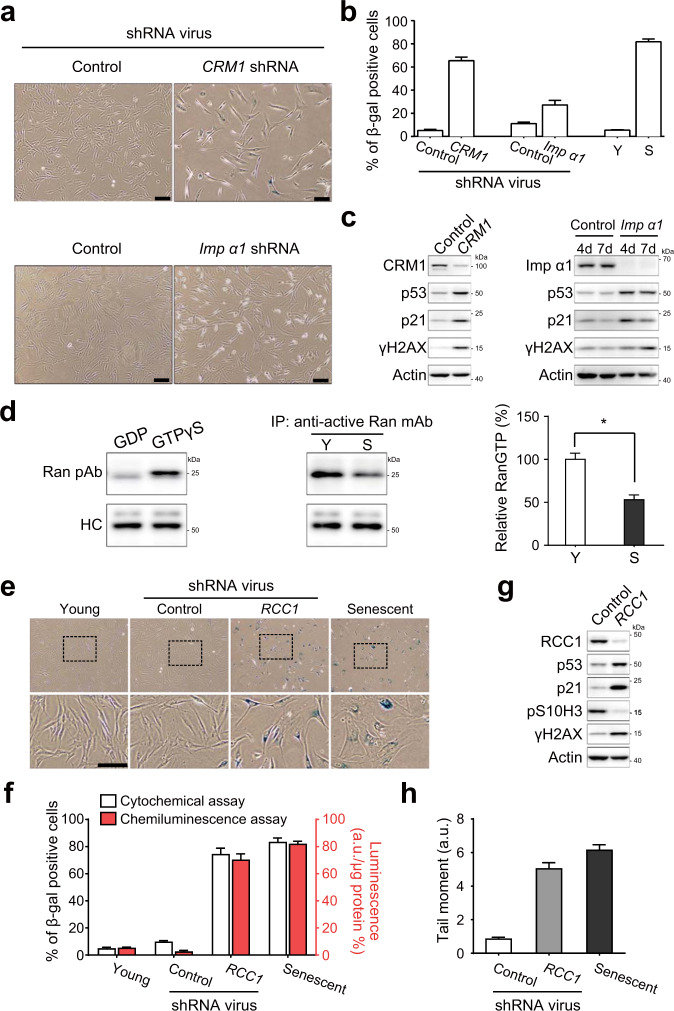
A genetically induced nuclear barrier results in RS-like senescence. **a** HDFs infected with lentivirus expressing CRM1 shRNA or importin-α1 shRNA for 9 days and treated with 1 μg/ml puromycin 2 days after infection. Representative images of cells stained for SA-β-gal activity by the cytochemical method. Scale bar, 50 μm. **b** Percentages of β-gal-positive cells in control and CRM1- or importin-α1-knockdown HDFs (*n* > 200 cells). Y and S, young and senescent HDFs, respectively. The β-gal assay was carried out 7 days after the start of puromycin selection. **c** Immunoblot of whole-cell lysates from HDFs infected with virus expressing CRM1 shRNA or importin-α1 shRNA. **d** Cell lysates immunoprecipitated with an anti-RanGTP antibody. As positive and negative controls, young cell lysates were used to pull down RanGTP after treatment with GDP or GTPγS. Immunoblotting of immunoprecipitates (IP) with an anti-Ran antibody. The right panel shows the quantitative analysis of RanGTP expression normalized to that of heavy chain. The results are representative of three independent experiments that are shown in the bar graph as the means ± SD. *, *P* < 0.05 (Student’s *t*-test). Y and S, young and senescent HDFs, respectively. **e** HDFs infected with lentivirus expressing RCC1 shRNA for 10 days. The cells were selected by treatment with 1 μg/ml puromycin 2 days after infection. Assays were performed 10 days after infection. Wide-field micrographs of cells stained for SA-β-gal activity by the cytochemical (X-gal) method. Bottom panels show 3× magnified versions of images as indicated by the dashed boxes on top. Scale bar, 50 μm. **f** Percentages of β-gal-positive cells among young, control shRNA-treated, RCC1-knockdown HDF, and RS HDF populations (Senescent) (*n* > 200 cells). Left (white) and right (red) bars indicate the percentages estimated by cytochemical and chemiluminescence assays, respectively. The data represent the means ± SD (*n* = 3); a.u., arbitrary unit. **g** Immunoblot using whole-cell lysates of HDFs infected with a lentivirus expressing control or RCC1 shRNA. **h** Comet assay with RCC1-knockdown HDFs and RS HDFs. The data represent the means ± SD (*n* > 50 cells).

We showed that a chemically induced nuclear barrier caused RS-like senescence in HDFs. However, the chemicals that induce this barrier may induce limited inhibitory effects^26,27^ or may exert potential off-target effects on nuclear transport^28^. Thus, we further confirmed the aforementioned findings using genetic inhibition of nuclear import and export (Supplementary Fig. S4a, b). Knockdown of *CRM1*, an exportin, or importin-α1, an importin, in HDFs using shRNAs significantly increased the percentage of SA-β-gal-positive HDFs (Fig. 3a, b). This increase was greater in the *CRM1*-knockdown HDFs than in importin-α1-knockdown HDFs and was comparable between *CRM1*-knockdown HDFs and HDFs undergoing RS, consistent with the results shown in Fig. 2b. Interestingly, we also found that depletion of either *CRM1* or importin-α1 induced DNA damage, as indicated by the increased levels of γH2AX (Fig. 3c).

Next, we examined whether genetic inhibition of the RanGTP gradient can induce RS-like senescence. We first confirmed that the level of RanGTP was significantly (*P* < 0.05) reduced in senescent cells compared to young cells (Fig. 3d), consistent with the gradual decrease in the protein levels of RCC1, a core enzyme involved in the generation of RanGTP, during RS (Fig. 1e). Knockdown of *RCC1* in young HDFs using shRNAs (Supplementary Fig. S4c) significantly increased the percentage of SA-β-gal-positive HDFs, which reached a level comparable to that in senescent cells (Fig. 3e, f). Loss of RCC1 also increased the levels of p53 and p21 expression but decreased the level of pS10H3 expression (Fig. 3g). After knockdown of *RCC1*, the extent of the DNA damage was increased in the young HDFs, as indicated by the increased levels of γH2AX and comet tail moments (Fig. 3g and h). Taken together, these data suggest that either chemically or genetically induced NBIS results in RS-like senescence phenotypes (cell cycle arrest and DNA damage).

### Aging-dependent disruption of NCT is observed in yeast

**Fig. 4:**
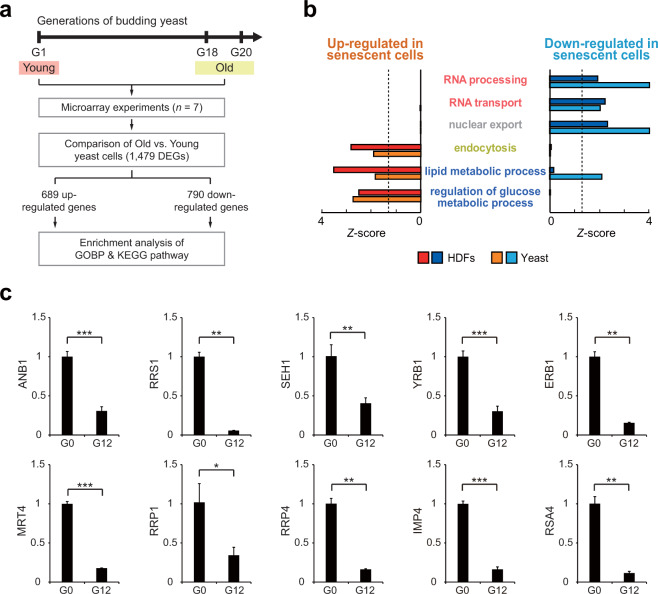
Disruption of NCT is conserved in aging yeast. **a** Schematic overview of the experimental design and microarray and data analyses. The number of DEGs between young and old yeast cells is presented. **b** Cellular processes (GOBPs) significantly (*P* < 0.1) enriched consistently by upregulated (red) or downregulated (blue) genes in aged yeast cells and HDFs undergoing RS. The Z-score indicates –N^−1^(*P*), where *P* is the enrichment *P* value determined with DAVID software and N^−1^(*·*) is the inverse normal distribution. **c** qRT-PCR analysis of representative genes involved in common NBIS-RS cellular pathways in the replicative aging yeast model. For the replicative aging model, the analysis was performed at 0 generations (young) and 12 generations (old). mRNA expression levels were first normalized to those of *CDC19* (internal control) and then further normalized by the mean mRNA expression level in young yeast cells. In this analysis, two additional non-DEGs, *SEH1* and *YRB1*, with *P* values close to the cutoff value, were included to confirm the downregulation of NCT. The values are presented as the means ± SD (*n* = 2 or 3). *, *P* < 0.05; **, *P* < 0.01; and ***, *P* < 0.001 by Student’s *t*-test.

We next examined whether disruption of NCT might be observed during the senescence of budding yeast, a single-cell eukaryotic organism with aging characteristics similar to those of cells in higher organisms^29^. To this end, we first obtained previously reported mRNA expression profile data (GSE10018) for young (1 generation) and old (18–20 generation) budding yeast cells (Fig. 4a). For this analysis, we used mRNA expression profiles because no proteomic data were available for evaluating the cytoplasmic and nuclear fractions of young and old yeast cells. Thus, for comparison, we performed mRNA expression profiling of young and senescent HDFs (RS). We identified 2388 differentially expressed genes (DEGs; 1328 upregulated and 1060 downregulated genes in senescent HDFs) between young and senescent HDFs and 1479 DEGs (689 upregulated and 790 downregulated genes in old yeast cells) between young and old yeast cells (Supplementary Table 3).

We next performed GOBP and KEGG pathway enrichment analyses of the up- and downregulated genes in senescent HDFs and old yeast cells. The enrichment analyses showed that the upregulated genes were significantly (*P* < 0.05) enriched in endocytosis and glucose and lipid metabolism in both senescent HDFs and old yeast cells. In contrast, the downregulated genes were enriched in NCT (nuclear export) and RNA processing and transport in senescent HDFs and old yeast cells (Fig. 4b). Next, we confirmed that the expression of the following 10 representative genes involved in NCT and RNA processing/transport was downregulated in two types of aging yeast models (replicative and chronological aging models) using quantitative real-time polymerase chain reaction (qRT-PCR) analysis: *ANB1, RRS1, SEH1*, and *YRB1*, which are involved in NCT, and *ERB1, MRT4, RRP1, RRP4, IMP4*, and *RSA4*, which are involved in RNA processing/transport (Fig. 4c and Supplementary Fig. S5). These data suggest that disruption of NCT, the formation of a nuclear barrier is evident in multiple eukaryote models of cellular aging.

### The senescence-associated changes in NBIS are most similar to those in RS

**Fig. 5:**
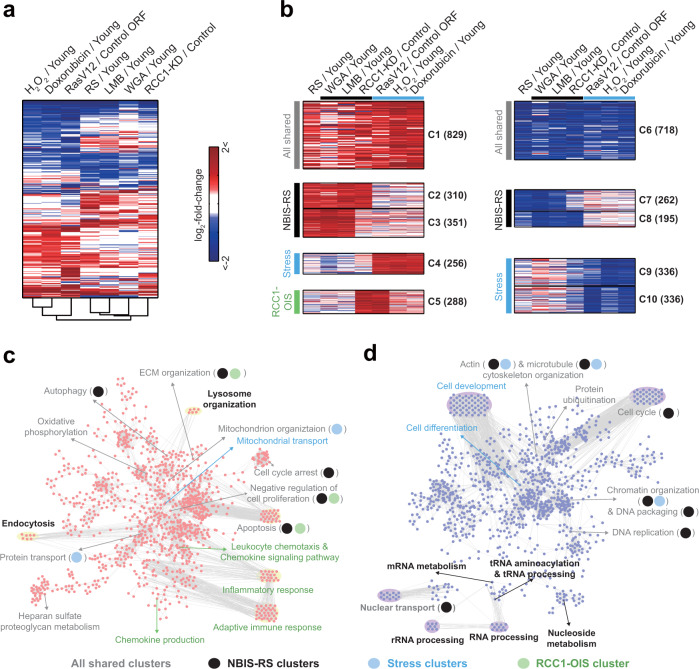
The senescence-associated changes in NBIS are most similar to those in RS. **a** Hierarchical clustering of seven cellular senescence models (RS-, WGA- and LMB-treated HDFs, RCC1-knockdown HDFs, OIS, OSIS, and DDIS) based on DEGs in at least one of the seven models (as determined by Euclidean distance and average linkage method). Colors represent the increase (red) and the decrease (blue) in mRNA expression levels for each senescence model with respect to its corresponding control. The color bar denotes the gradient of log_2_-fold-change between each senescence model and its corresponding control. **b** Top 10 clusters of DEGs identified by NMF clustering analysis based on their differential expression patterns across the seven cellular senescence models. The color scheme is the same as that shown in (**a**). **c**, **d** Process network models constructed for upregulated genes in C1-5 (**c**) and downregulated genes in C6-10 (**d**) using cellular process association analysis. Magenta and purple nodes represent GOBPs/KEGG pathways significantly (*P* < 0.1) enriched with genes in at least one of the upregulated (C1-5) and downregulated clusters (C6-10). Edges represent significant genes overlap between the connected GOBPs/KEGG pathways. Label colors or circle colors indicate that the corresponding GOBPs/KEGG pathways are enriched by the genes in the corresponding clusters (see legend at the bottom of the figures). Circles are used only for a GOBP/KEGG pathway enriched by two or more types of clusters, including ‘All shared clusters’.

To compare NBIS with other senescence models, we further performed mRNA expression profiling of OIS (RasV12-overexpressing HDFs), OSIS (H_2_O_2_-treated HDFs), and DDIS (doxorubicin-treated HDFs) models, as well as NBIS models including LMB- and WGA-treated HDFs and RCC1-knockdown (RCC1-KD) HDFs. We then identified DEGs in each senescence model. For combining DEGs from all senescence models (Supplementary Table 4), we performed hierarchical clustering of the DEGs to examine the similarity in senescence-associated expression changes among the senescence models. The NBIS models (LMB-treated, WGA-treated, and RCC1-KD models) clustered with the RS model. Among the NBIS models, the LMB-treated model was grouped first with the RS model, consistent with the finding shown in Fig. 2h. On the other hand, the OIS and stress-induced premature senescence models (OSIS and DDIS models) clustered separately (Fig. 5a).

To compare the expression changes in these senescence models, we next performed non-negative matrix factorization (NMF) clustering of the DEGs using their log_2_-fold-change values as previously described^30^ (Fig. 5b). Among the resulting clusters, we focused on the top 10 clusters: C1 and C6 included up- and downregulated genes shared among all the models, respectively (‘all shared cluster’); C2-3 and C7-8 included up- and downregulated genes predominantly common to the NBIS and RS models, respectively (‘NBIS-RS clusters’); C4 and C9-10 included up- and downregulated genes predominantly evident in the stress-induced senescence models (‘stress clusters’); and C5 included upregulated genes that were also upregulated in the RCC1 shRNA-transfected senescence and OIS models (‘RCC1-OIS cluster’). Of the top 10 clusters, six (the all shared and NBIS-RS) clusters included genes that exhibited senescence-associated expression changes also observed in the RS model. Taken together, these data suggest that among the various senescence models tested, the NBIS model is most similar to the RS model in terms of senescence-associated expression changes.

To identify the cellular processes associated with the DEGs, we performed GOBP and KEGG pathway enrichment analysis of the DEGs in C1-10. To examine the relationships among the enriched GOBPs/KEGG pathways, we generated two network models describing the relationships (edges) among the GOBPs and KEGG pathways (nodes) enriched with genes in the upregulated (C1-C5) and downregulated clusters (C6-C10). In the upregulated network, the ‘all shared cluster’ (C1) genes were associated with processes related to 1) cell cycle arrest and apoptosis; 2) mitochondrial organization and oxidative phosphorylation; 3) protein transport; 4) autophagy; 5) extracellular matrix (ECM) organization; and 6) proteoglycan metabolism, suggesting that these processes were consistently promoted in all the senescence models (Fig. 5c). ‘NBIS-RS cluster’ (C2-3) genes were associated with endocytosis and lysosome organization, as well as cell cycle arrest (Fig. 5c). These data suggest that upregulation of cell cycle arrest and endocytic and lysosomal networks may play important roles in the NBIS and RS models. On the other hand, the ‘stress cluster’ (C4) and ‘RCC1-OIS cluster’ (C5) genes were predominantly associated with mitochondrial protein transport and immune response-related processes.

In the downregulated network, the ‘all shared cluster’ (C6) genes were associated with processes related to 1) NCT; 2) the cell cycle (chromatin organization and DNA replication); and 3) protein ubiquitination. ‘NBIS-RS cluster’ (C7-8) genes were associated with processes related to RNA processing/transport and nucleoside metabolism, as well as NCT and the cell cycle (Fig. 5d). These data suggest that the downregulation of NCT, the cell cycle, RNA processing/transport, and nucleoside metabolism may play important roles in the NBIS and RS models. On the other hand, ‘stress cluster’ (C6-7) genes were predominantly associated with differentiation-related processes (regulation of cell differentiation and cell development). Notably, the processes related to shared up- and downregulated genes in the NBIS and RS models were largely consistent with those identified by proteomic analysis (Fig. 1d) and conserved in aged yeast (Fig. 4b). Taken together, these data suggest the potential importance of these processes common to both NBIS and RS in physiological cellular senescence.

### Processes common to NBIS and RS coordinate the reduction of NCT

**Fig. 6:**
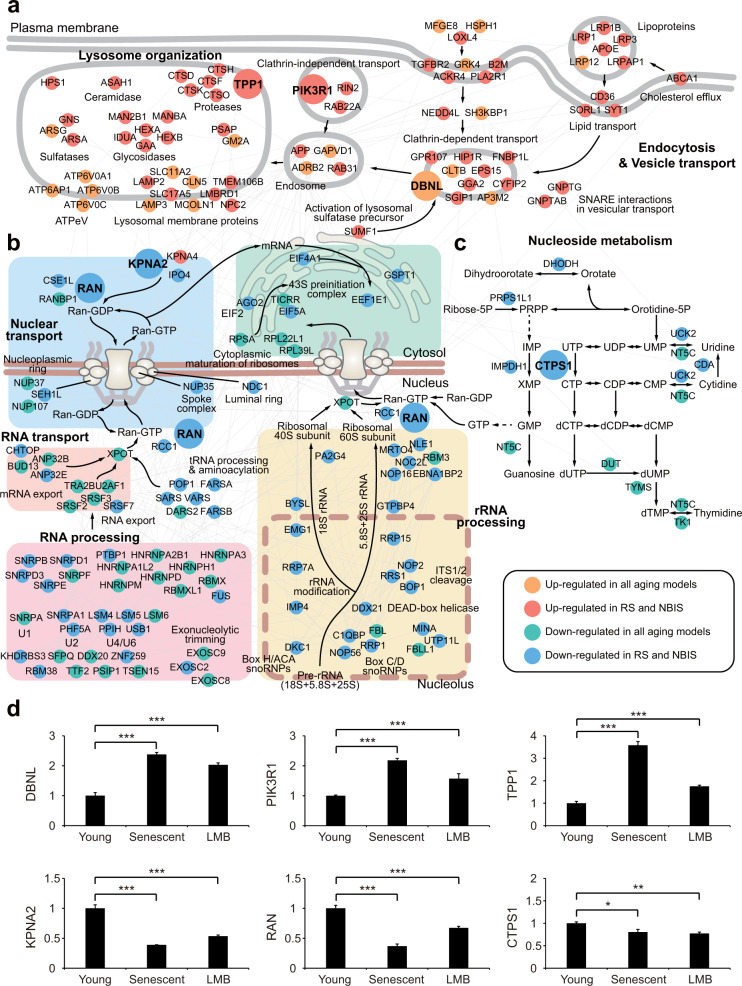
Processes common to NBIS and RS coordinate the reduction of NCT. **a**–**c** Network models showing interactions among DEGs involved in endocytosis and lysosomal degradation (**a**), nuclear transport and RNA processing (**b**), or nucleotide metabolism (**c**). Node colors represent the clusters to which the corresponding belong (see legend box). Gray lines denote protein-protein interactions, arrows represent the transport of molecules or metabolic reactions, the dotted line denotes the membrane of the nucleolus, and the two thick lines represent the plasma or nuclear membrane. Names of complexes or functional modules for the closely located genes are shown. **d** qRT-PCR analysis of representative genes in the cellular pathways common to both NBIS and RS. The analyzed genes are shown as large nodes in the network models (**a**–**c**). mRNA expression changes were first normalized to those of *RPS11* (internal control) and then further normalized by the mean mRNA expression change in young HDFs. The values are presented as the means ± SD (*n* = 2 or 3). *, *P* < 0.05; **, *P* < 0.01; and ***, *P* < 0.001 by one-way analysis of variance with Dunnett’s post hoc test.

We next generated network models describing the interactions among gene products involved in processes common to NBIS and RS according to protein-protein interactions (endocytic and lysosomal network, NCT, and nucleoside metabolism). The endocytic and lysosomal network (Fig. 6a) showed coordinated upregulation of 1) ‘endocytosis’ pathways, including clathrin-dependent (*CLTB*, *EPS15*, *SGIP1*, *GGA2*, *GRP107*, *DBNL*, *FNBP1L*, *CYFIP2*, and *HIP1R*) and clathrin-independent (*PI3KR1*, *RIN2*, and *RAB22A*) pathways and lipid/lipoprotein transport pathways (*LRP1/1B/3/12*, *LRPAP1*, *APOE*, *CD36*, *SORL1*, and *SYT1*), and 2) lysosomal degradation pathways of endocytosed proteins and lipids, including ceramidase (*ASAH1*), proteases (*CTSD/F/H/K/O* and *TPP1*), glycosidases (*MAN2B1*, *MANBA*, *IDUA*, *HEXA/B*, and *GAA*), vacuolar ATPases (*ATP6V0A1/B/C* and *ATP6AP1*), and lysosomal membrane proteins (*LAMP2/3* and *SLC11A2/17A2*). This orchestrated upregulation may reduce the transmission of extrinsic signals to the nucleus.

The NCT network (Fig. 6b) illustrated coordinated downregulation of (1) the ‘NCT system’ (e.g., *RAN*, *RCC1*, *CSE1L*, and *RANBP* in the RanGTP gradient), (2) ‘RNA export’ pathways (e.g., *SRSF2/3/7*, *TRA2B*, and *U2AF1* for RNA; *ANP32B/E*, *BUD13*, and *CHTOP* for mRNA), (3) ‘RNA processing’ pathways including spliceosome components (e.g., *SNRPB/D1/D3/E/F* and *HNRNPA1L2/A2B1/A3/D/H1/M*) and splicing factors (*SFPQ* and *PSIP1*), and (4) rRNA processing pathways for ribosome assembly (*DKC1*, *IMP4*, *EMG1*, *DDX21*, *RRP1/7* *A/15*, *FBL/L1*, *RRS1*, and *BOP1*). These pathways are involved in the supply of proteins and RNAs produced in the nucleus to the cytoplasm. Their orchestrated downregulation may reduce the supply of materials trafficked out of the nucleus. Moreover, the nucleoside metabolism network (Fig. 6c) showed downregulation of metabolic pathways (*UCK2*, *NT5C*, *CDA*, *CTPS1*, *DHODH*, *PRPS1L1*, *IMPDH1*, *DUT*, *TYMS*, and *TK1*) that are linked to the production of GTP, and therefore the GDP/GTP ratio is maintained, which may explain the reduced supply of GTP available for the RanGTP gradient, resulting in disruption of the RanGTP gradient-dependent NCT system.

Finally, qRT-PCR analysis confirmed similar changes in expression of the following representative genes during NBIS and RS (Fig. 6d): (1) *DBNL*, *PIK3R1*, and *TPP1*, which are involved in endocytic and lysosomal networks; (2) *KPNA2* and *RAN*, which are involved in NCT; and (3) *CTPS1*, which is involved in nucleoside metabolism. These networks collectively suggest decreased (1) transmission of extrinsic signals to the nucleus because of increased lysosomal degradation and (2) supply of materials from the nucleus to the cytoplasm because of substantially inhibited nuclear transport in both the NBIS and RS models. In summary, dysregulation of both endocytic and lysosomal networks and NCT may be critical for the RS-like physiological changes observed in NBIS. These data collectively suggest that the formation of a nuclear barrier is as a key event in cellular senescence.

### Knockdown of the Sp1 transcription factor downregulates the expression of NCT-related genes

**Fig. 7:**
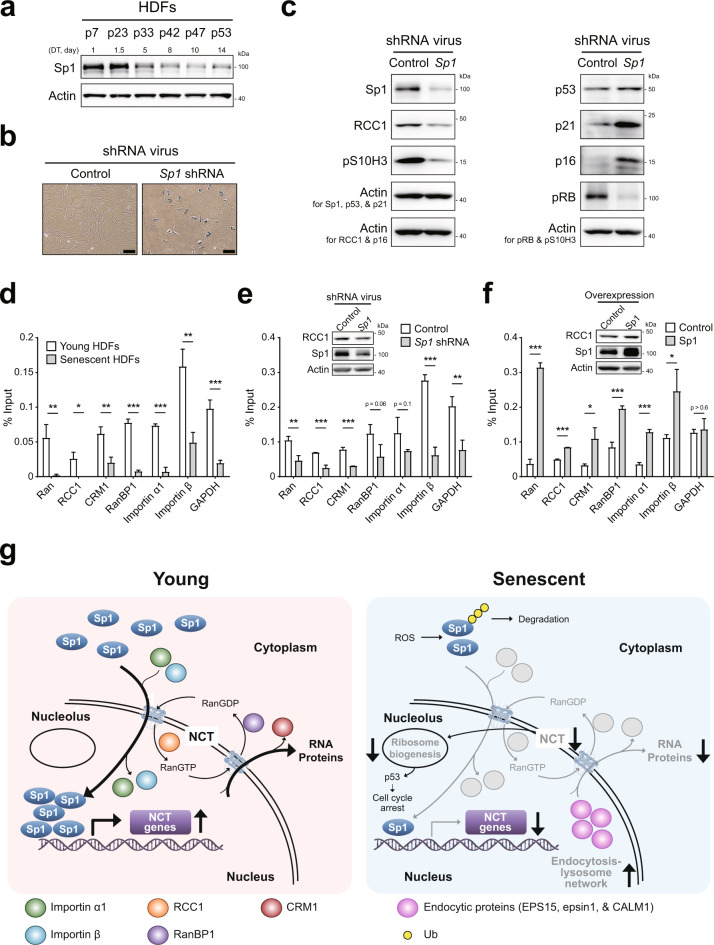
The expression of NCT regulators is regulated by Sp1 expression. **a** Immunoblot of whole-cell lysates of HDFs at different passages while undergoing senescence. Doubling time (DTs) corresponding to passage number are shown. **b** HDFs were infected with a lentivirus expressing Sp1 shRNA for 14 days; cells were selected by treatment with 5 μg/ml puromycin starting 2 days postinfection. Wide-field micrographs of cells stained for SA-β-gal activity by the cytochemical (X-gal) method. Scale bar, 50 μm. **c** Immunoblot of whole-cell lysates of HDFs infected with a lentivirus expressing Sp1 shRNA. **d** ChIP analysis with an anti-Sp1 antibody using young or senescent HDFs. The binding of Sp1 to the gene promoter was assessed by qPCR. The results are representative of three independent experiments; histograms show the means ± SD (*n* = 3). *, *P* < 0.05; **, *P* < 0.01; and ***, *P* < 0.001 by Student’s *t*-test. **e** Immunoblot and ChIP analyses with an anti-Sp1 antibody using HDFs infected with a lentivirus expressing Sp1 shRNA or control shRNA. The binding of Sp1 to the gene promoter was assessed by qPCR. The details are same as those shown in (**d**). **f** Immunoblot and ChIP analyses with an anti-Sp1 antibody using HDFs transfected with Sp1 expression or empty vector. The details are same as those shown in (**d**). **g** Proposed mechanistic model of NBIS.

We previously reported that the expression levels of nucleoporins (Nups) and their associated nuclear pore complexes are decreased during senescence^10^ and that a senescence-dependent decrease in the expression of the Sp1 transcription factor is critical for the downregulation of Nup expression^15^. We thus hypothesized that the senescence-dependent decrease in Sp1 expression may contribute to the downregulation of the expression of NCT-related genes, thereby leading to the establishment of a nuclear barrier. To test this hypothesis, we first measured the protein levels of Sp1 in HDFs at different passages during RS. The protein levels of Sp1 were found to be decreased after passage 33 of cells undergoing RS (Fig. 7a). We then examined the effect of Sp1 on cellular senescence, the cell cycle, and cell proliferation. Knockdown of Sp1 using optimal shRNAs (Supplementary Fig. S4d) increased SA-β-gal activity (Fig. 7b) and the expression levels of p53, p21, and p16 (Fig. 7c). In contrast, Sp1 knockdown decreased the expression levels of pS10H3 and pRb (Fig. 7c). These data indicate that downregulation of Sp1 expression can lead to cellular senescence.

We next sought to determine whether Sp1 can regulate the expression of NCT-related genes. Chromatin immunoprecipitation (ChIP) analysis using sheared chromatin obtained from young and senescent HDFs showed that Sp1 bound to the promoters of importins (α1 and β), Ran, RanBP1, CRM1, and RCC1 and that the binding strengths decreased as the HDFs senesced (Fig. 7d). Knockdown of Sp1 expression in young HDFs resulted in the same decrease in binding to promoters of NCT-related genes (Fig. 7e), which caused downregulated expression of RCC1 (Fig. 7c, e). On the other hand, overexpression of Sp1 in young HDFs increased binding to promoters of these NCT-related genes (Fig. 7f). Together with the previous finding of the aging-dependent downregulation of Sp1 expression, these data suggest a mechanistic model for NBIS (Fig. 7g). In this model, an aging-dependent decrease in Sp1 expression may be critical for the downregulation of the expression of NCT-related genes, thereby leading to the formation of a nuclear barrier. The model also shows previously reported regulatory links of downregulated NCT to promotion of the endocytosis-lysosome network and inhibition of ribosome biogenesis.

## Discussion

NCT is intimately associated with the regulation of cellular functions in the nucleus, such as the cell cycle, DNA replication/repair, chromatin remodeling, and transcriptional activation. These events are also ATP-driven and energy-consuming processes. As cells undergo senescence, these events are slowed or halted in a time-dependent manner. However, how senescent cells coordinate these complicated processes effectively is unclear. In this study, we showed that this process can be initially activated by the simultaneous shutdown of energy-consuming nuclear events and NCT through the formation of a nuclear barrier. Integrative proteomic and transcriptomic analysis showed that dysregulation of endocytic and lysosomal networks and NCT in senescent cells results in the coordinated reduction in the transmission of extrinsic signals to the nucleus and the supply of proteins and RNAs trafficked from the nucleus. This feature was also observed in aging yeast, implying that nuclear barrier formation is a characteristic of physiological aging in all eukaryotes.

Our integrative analysis of proteomic and transcriptomic data showed consistent alterations in cellular processes in RS and NBIS: upregulation of endocytic and lysosomal networks and mitochondrial organization and downregulation of NCT and the cell cycle/DNA replication. In addition, the proteomic data provided further information complementary to transcriptomic data indicating the location of these alterations in either the nucleus or cytoplasm. This additional information suggests functional relationships among these processes: Dysregulation of NCT may induce accumulation of proteins involved in endocytic and lysosomal networks and mitochondrial organization, resulting in their upregulation in the nucleus. Moreover, the proteomic analysis revealed senescence-associated nuclear enrichment of several cellular processes that do not occur in the nucleus under normal conditions. For example, the levels of proteins involved in glucose, fatty acid metabolism, and mitochondrial organization were elevated in the nucleus during senescence. According to previously described canonical and noncanonical nuclear functions of these proteins^31^, their enrichment in the nucleus suggests the establishment of an energy supply in the nucleus (canonical) during senescence involving mitochondrial dysregulation or utilization of these proteins for nuclear signaling or transcriptional regulation to counteract the reduction in signal transduction during senescence; this speculation should be further tested using detailed experiments. On the other hand, a comparative transcriptomic analysis of seven senescence models indicated that NBIS-specific alterations of cellular processes are also common to RS. Thus, our integrative analysis of proteomic and transcriptomic data afforded a more reliable and comprehensive understanding of senescence-associated changes mediated by the nuclear barrier.

In this study, NBIS induced by WGA (WGA-NBIS) resulted in weak reversible senescence responses (SA-β-gal activity and DNA damage). In contrast, NBIS induced by LMB (LMB-NBIS) resulted in stronger irreversible senescence responses than those induced by WGA-NBIS. Correspondingly, cells undergoing LMB-NBIS showed more RS-like gene expression changes than those undergoing WGA-NBIS. Nuclear import enables extrinsic signal transmission to the nucleus that allow adaptation of the transcriptional program to environmental alterations, and nuclear export supplies proteins and RNAs from the nucleus to the cytoplasm, where they can execute their cellular functions according to the transcriptional program. Our results suggest that impaired nucleus-to-cytoplasm transport of proteins and RNAs might be more effective for inducing irreversible senescence phenotypes than reversible disruption of the transmission of extrinsic signals to the nucleus. One of the shared features of NBIS and RS is the upregulation of endocytic and lysosomal degradation of proteins and lipids, which may compensate for the reduction in nuclear signal transmission. Notably, differential effects of WGA and LMB on senescence cannot be ruled out, and the weaker senescence phenotypes observed after WGA treatment might have been partially the result of inadequate inhibition or off-target effects.

Conventional senescence models (RS, OIS, OSIS, and DDIS models) were previously shown to exhibit shared features, including increased SA-β-gal activity and DNA damage^32^. However, differences between RS models and other senescence models have also been reported^33,34^. For example, comparative proteomic analyses of OSIS and RS models have revealed a significant number of differentially regulated proteins in cells undergoing RS and OSIS^34^. Moreover, the expression of *hTERT* has a senescence-bypassing effect in RS but not in OIS^33^. In this study, we compared the genome-wide gene expression profiles in the RS, OIS, OSIS, and DDIS models. The RS model showed senescence-associated gene expression patterns that were distinct from those of the OIS, OSIS, and DDIS models. In contrast to RS, OIS was predominantly associated with inflammatory and adaptive immune responses, while OSIS and DDIS were associated with cell development. In contrast, there was a considerable overlap of senescence-associated gene expression patterns between the NBIS and RS models, such as downregulated expression of genes related to NCT and upregulated expression of genes related to endocytosis and lysosome networks, indicating the significance of these pathways in physiological cellular senescence. Interestingly, although NBIS indirectly induces DNA damage (Fig. 2d) putatively via p53-related apoptosis^35^ or RNA GTPase-associated ROS^36^, the NBIS model in our study showed different mRNA expression alteration patterns than the DDIS model, in which DNA damage was directly induced by doxorubicin (C7-8 and C9-10 in Fig. 5b). Due to the direct damage mechanism, DDIS results in more severe DNA damage than NBIS. The difference in the damaging mechanism and severity may cause differential mRNA expression alteration patterns in NBIS and DDIS, as indicated by different sets of genes with expression levels that were specifically downregulated by NBIS (POLD2) and DDIS (REV3L, RMI2, and CUL4B).

Moreover, the pathological implications of NCT have been observed in several diseases, such as cancers, metabolic disorders, and infectious diseases^37^. For example, CRM1 expression is highly upregulated in various types of cancers, such as rectal cancer, osteosarcoma, and ovarian cancer^38–40^. Mutations in the mRNA export mediator *GLE1* cause human lethal congenital contracture syndrome-1^41^. Disruption of the Ran gradient in pathological aging in Hutchinson-Gilford progeria syndrome has been reported^16^. We recently found global transcriptional downregulation of mRNA export (TREX) and nuclear pore components in human aging^42^. Our current study further demonstrated the utility of the nuclear barrier in the NCT system as a potential indicator of or therapeutic target for cellular senescence. For example, the levels of nuclear transport components (CRM1, RCC1, importin-α/βs, and RanGTP/GDP) can be used as useful indicators for evaluating the state of cellular aging. However, whether these mechanisms of senescence are physiologically relevant in vertebrate systems in vivo remains to be studied.

As various cellular stages from early RS to late RS have been proposed^43^, determining whether nuclear barrier formation occurs prior to other stress-dependent changes by monitoring nuclear barrier formation may be an efficient strategy for assessing the initiation and progression of early RS. Recently, reversal of early RS by cell physiology modulators^44,45^ or selective senolytic induction of senescent cell death^46,47^ has recently been applied as a potential therapeutic approach to aged tissues. In this context, the nuclear barrier observed prior to early RS may serve as a promising therapeutic target for modulation of cellular senescence or as a target for a senolytic strategy, which would provide an alternative approach for the functional recovery of aged cells or tissues. In addition, our data may contribute to the development of an efficient and time-saving method to study physiological cellular senescence and its underlying mechanism through use of the NBIS model as a substitute for the RS model, which requires long-term cell culture.

Our mechanistic model of NBIS shows both upstream and downstream pathways of NCT. Aging-dependent downregulation of Sp1 transcription factor expression is a potent upstream factor that can cause inhibition of NCT during senescence. We previously demonstrated that Sp1 overexpression prevents TRF2ΔBΔM-induced premature senescence, as indicated by increased and decreased percentages of BrdU- and SA-β-gal-positive cells, respectively, as well as increased expression of NCT genes, including importin α, Nup107, and Nup50^48^. Several downstream pathways of NCT linked to (1) the endocytosis-lysosome network and (2) ribosome biogenesis are suggested based on the following previous findings: (1) LMB-induced NCT impairment induces the accumulation of endocytic proteins (e.g., EPS15, epsin1, and CALM1) in the nucleus^49^; and (2) impairment of NCT causes the accumulation of stress in the nucleolus^50^, a center for ribosome biogenesis, which can trigger p53 activation leading to cell cycle arrest^51^. However, further details of these mechanisms need to be determined. In summary, we identified a novel senescence trigger, nuclear barrier formation, which might serve as a potential indicator or therapeutic target of cellular senescence and provide new insights into the molecular mechanisms involved in the physiological aging of eukaryotes. Therefore, we propose that NBIS is a new modality of cellular senescence and that the NBIS can be used as an aging model to study physiological senescence.

## Materials and methods

### Cell culture, treatment, and reagents

Normal neonatal HDFs (PCS-201-010, American Type Culture Collection) were cultured in Dulbecco’s modified Eagle’s medium (1×) with 10% fetal bovine serum (Welgene) containing 100 U/ml penicillin and 100 μg/ml streptomycin (Welgene). The cells were grown at 37 °C and 5% CO_2_. The cells were treated with 0.1 μg/ml leptomycin B (LMB, Sigma) to inhibit nuclear export or with 6 μg/ml wheat germ agglutinin (WGA, Sigma) to inhibit nuclear import. Cells were serially passaged, and young proliferating and senescent cells were collected at doubling times (DTs) ≤ 1 day and ≥ 14 days, respectively.

### Lentiviral and retroviral vectors and viral transduction

The RNA Consortium (TRC) lentiviral human CRM1 shRNA (RHS4533-EG7514), human importin-α1 (KPNA2) shRNA (RHS4533-EG3838), and human RCC1 shRNA (RHS4533-EG1104) were obtained from Dharmacon; pLenti CMV/TO RasV12 Puro (w119-1) was purchased from Addgene (Plasmid #22262), and pLenti6/U6 NRMT shRNA (5’-AGAGAAGCAATTCTATTCCAAG-3’) was subcloned using a pLenti6/U6 vector. Viruses were produced using protocols recommended in the manufacturer’s instructions (Invitrogen). Briefly, for lentivirus expression, 293FT human embryonic kidney cells (#R700-07; Invitrogen) were transfected with the expression vector and ViraPower packaging mix (pLP1, pLP2, and pLP/VSVG) using Lipofectamine 2000 (Invitrogen). For retrovirus expression, GP2-293 cells (631458; Clontech) were transfected with the expression vector and pVSV-G envelope vector using Lipofectamine 2000. The supernatant containing virus was collected 2 or 3 days after transfection, concentrated 10-fold using Lenti-X (631231) and Retro-X Concentrator (631455) (Clontech), flash-frozen in liquid nitrogen, and stored at −80 °C. On the day of transduction, an aliquot of the viral stock was thawed on ice and added to growth medium containing polybrene (4 and 6 μg/ml for the retro- and lentiviral infection, respectively). Cells were cultured in virus-containing medium overnight, washed with Dulbecco’s phosphate-buffered saline (PBS), and returned to culture in normal growth medium.

### Induction of cellular senescence

For OIS, young HDFs (5 × 10^5^) were infected with RasV12-expressing lentivirus at an MOI of 2. For DDIS, young HDFs were treated with 0.5 μM doxorubicin for 1 day and then grown in medium without drugs for 7 days. For OSIS, young HDFs were treated with 250 μM H_2_O_2_ for 1 day and then grown in medium without drugs for 7 days.

### Senescence-associated β-gal activity assay

The SA β-gal assay was carried out with X-gal staining^52^ or quantitative chemiluminescence using a modified version of the Galacto-Light system^53^. For the latter procedure, cells were seeded in 6-well plates, washed twice with PBS for 2 days, and then lysed in 50 μl of cell culture lysis buffer; 5 μl of the lysate was incubated with Galacton substrate in 200 μl of reaction buffer (100 mM sodium phosphate, pH 6.0, and 20 μM MgCl_2_) for 40 min at room temperature, and 300 μl of Emerald luminescence amplifier was added immediately before the luminescence was measured on a luminometer. The results were normalized by the amount of proteins present in the same sample. Protein concentrations were determined by Bradford assay.

### Antibodies

We purchased anti-p53 (sc-126), anti-CRM1 (sc-5595), anti-RCC1 (sc-1162), anti-RanBP1 (sc-1160), anti-Myo6 (sc-393558), anti-EPLIN (sc-136399), and anti-HEXB (sc-376781) antibodies from Santa Cruz Biotechnology; anti-p21 (#554228), anti-Rb (#554136), anti-importin-β (#610560), and anti-Ran (#610340) antibodies from BD Biosciences; anti-p16 (#1963-1) and anti-RCC1 (#5134) from Epitomics; anti-pS10H3 (#9701), anti-γH2AX (#9718), anti-caspase-3 (#9665), anti-CALD1 (#12503), anti-CTSD (#2284), anti-MEK (#9122), anti-ADRM1 (#12019), anti-HSPD1 (#12165), and anti-HSP90B1 (#2104) from Cell Signaling Technology; anti-importin-α1 (NB100-1371) from Novus Biologicals; anti-actin (A1978) from Sigma; anti-53BP1 (#05-726) and anti-trimethylated-SPK (#07-1814) from Millipore; and anti-PSMB4 (AB137067), anti-NRMT (AB72660) and anti-LMNA (AB26300) from Abcam.

### RanGTP activation assay

RanGTP levels were measured using Ran activation assay kits obtained from Abcam (AB173247) or Cell Biolabs (#STA-409) according to the manufacturer’s instructions. For use with the former kit, a configuration-specific monoclonal antibody that specifically recognizes RanGTP but not RanGDP is used, while for use in the latter kit, RanBP1-agarose beads are used to selectively pull down RanGTP. Briefly, 5 × 10^6^ cells were lysed in 100 μl of 1× assay/lysis buffer containing cOmplete protein inhibitor cocktail (Roche) and PhosSTOP (Roche). Lysates were centrifuged at 14,000 × *g* for 10 min at 4 °C and then incubated on a rotator for 1 h at 4 °C with RanGTP antibody/protein A/G agarose or RanBP1 agarose beads, which were then separated from the supernatant by centrifugation at 14,000 × *g* for 1 min and washed four times with 1× assay/lysis buffer. The beads were resuspended in 2× SDS sample buffer, heated at 95 °C for 5 min, and the associated proteins were then resolved by SDS polyacrylamide gel electrophoresis.

### Chromatin-immunoprecipitation assay

Quantitative ChIP was performed as previously described^54^. Briefly, 1 × 10^7^ cells were treated with 1% formaldehyde for 10 min at 37 °C. Chromatin was fragmented on ice by sonication with four 30-s pulses delivered from a Bioruptor (Cosmo Bio). Chromatin was precleared by incubation with protein A or G magnetic beads (Life Technologies). After the beads were removed, the chromatin was incubated overnight with specific or isotype control antibodies at 4 °C, followed by incubation with protein A or G magnetic beads for 1 h at 4 °C. Bound protein-DNA complexes were eluted after washing. DNA was isolated by phenol-chloroform extraction. A portion of recovered DNA from each ChIP experiment was evaluated by qPCR using the primers listed in Supplementary Table 5.

### Neutral comet assay

A neutral comet assay was performed using a single-cell gel electrophoresis assay kit (Trevigen) according to the manufacturer’s instructions with minor modifications. Briefly, 1 × 10^5^ cells were diluted in 0.5 ml of ice-cold PBS, and a 50-μl cell suspension was resuspended in 500 μl of LMAgarose and rapidly spread onto slides. DNA was stained with SYBR-gold (Life Technologies), and olive tail moments (expressed in arbitrary units) were calculated by counting from 100 to 200 cells per condition. The data were analyzed with Metafer4 software (MetaSystems). Bar graphs were plotted using GraphPad Prism v8.0 software (GraphPad Inc.) with the standard error of the mean. Statistical analysis was performed using the nonparametric Mann-Whitney U rank sum *t*-test in GraphPad Prism v.8.0.

### Subcellular fractionation

To isolate the cytoplasmic and nuclear fractions, cells were lysed in buffer A (10 mM HEPES [pH 8.0], 1.5 mM MgCl_2_, 10 mM KCl, 0.5 mM DTT, 300 mM sucrose, 0.1% NP40, 0.5 mM PMSF) and incubated for 5 min on ice. The samples were centrifuged at 10,000 rpm at 4 °C for 1 min, and the supernatant was collected as the cytoplasmic fraction. The pellet was resuspended in buffer B (20 mM HEPES [pH 8.0], 20% glycerol, 100 mM KCl, 100 mM NaCl, 0.2 mM EDTA, 0.5 mM DTT, and 0.5 mM PMSF) and incubated for 15 min on ice. The samples were centrifuged at 12,000 rpm at 4 °C for 5 min, and the supernatant was used as the nuclear fraction.

### LC-MS/MS analysis

Proteins were isolated from cytoplasmic and nuclear fractions and subjected to tryptic digestion. The resulting peptides were labeled using TMT reagents, and the labeled peptide samples were fractionated into 24 fractions as described in the Supplementary Methods. Twenty-four prepared fractions of peptide samples were then analyzed on a Q Exactive Orbitrap mass spectrometer coupled online with an EASY nLC 1000 system (Thermo Fisher Scientific, Bremen, Germany). A PepMap^TM^ RSLC C18 (75 μm × 50 cm, 2 μm) column and Acclaim PepMap^TM^ 100 nanoViper C18 (75 μm × 2 cm, 3 μm) column were used as the analytical column and trap column, respectively. Solvent A consisted of 2% acetonitrile and 0.1% formic acid in water, and Solvent B consisted of 2% water and 0.1% formic acid in acetonitrile. Peptides in a mixture were loaded onto the trap column and then separated on the analytical column with a 120-min linear gradient (0-5 min, 5% solvent B; 5-85 min, a shallow linear increase from 5 to 25% solvent B; 85-95 min, a sharp increase from 25 to 90% solvent B; 95–105 min, 90% solvent B; 105–110 min, 90 to 5% solvent B; and 110–120 min, 5% solvent B). The column flow rate was 300 nL/min, and the temperature of the column was set at 50 °C. Full MS scans were acquired at 35,000 resolution with the auto gain control target value of 1.0 × 10^6^ and the maximum ion injection time of 100 milliseconds. A total of the 20 most abundant ions were subsequently isolated and subjected to gas phase dissociation using higher-energy collision dissociation (HCD)^55^ with a normalized collision energy of 29%. The dynamic exclusion value was set to 30 s. MS/MS scans were acquired at 70,000 resolution with an automatic gain control value of 1.0 × 10^5^. Lock mass from ambient air (m/z 445.12003) was used for the internal calibration^56^. The raw data were deposited in the Proteomics Identifications (PRIDE) database with an accession ID, PXD025618.

### Identification of DEPs

Peptides were identified and quantified using the datasets generated from the LC-MS/MS analysis results, as described in the Supplementary Methods. To identify DEPs in the nucleus or cytoplasm, we first identified DE peptides. Briefly, we normalized reporter ion intensities of identified peptides using quantile normalization^57^ and selected peptides unique to a protein group and that had precursor isolation purities > 75% and maximum log_2_-intensities across TMT channels > the 5^th^ percentile of the log_2_-intensity distribution. For individual peptides in the nucleus or cytoplasm, we computed *T* values by Student’s *t*-test and log_2_-median-ratios between senescent HDFs (or LMB-treated young HDFs) and young HDFs. We then generated empirical null distributions of the *T* values and log_2_-median-ratios by performing random permutations of the TMT channels 100 times. Next, the adjusted *P* values were calculated by applying a two-tailed test to the observed *T* values using the empirical distribution^58^. The DE peptides were identified as those with *P* values < 0.05 and absolute log_2_-median-ratios > the cutoff, as determined by the mean absolute values of the 5^th^ and 95^th^ percentiles in the empirical distribution of log_2_-median-ratios. Additionally, we determined that the DE peptides were to be identified under only one condition (> more than two replicates). DEPs in the nucleus and/or cytoplasm were then determined as described in the Supplementary Methods. Finally, we performed enrichment analyses of GOBPs and KEGG pathways for the DEPs in the nucleus and cytoplasm using DAVID software^59^. GOBPs and KEGG pathways enriched by the genes in each cluster were identified as those with *P* < 0.1 and ≥5 genes.

### Microarray experiments

Total RNA was isolated from RS HDFs, young HDFs and young HDFs treated with LMB, WGA, RCC shRNA, H_2_O_2_, doxorubicin, or RasV12. RNA purity and integrity were evaluated with an ND-1000 spectrophotometer (NanoDrop) and Bioanalyzer 2100 (Agilent), respectively. The average RNA integrity number of the samples was 9.4. The RNA was amplified and labeled with Cy3-dCTP according to the standard Agilent One-Color microarray protocol (Agilent, V6.5, 2010). The amplified cRNA was hybridized to Agilent SurePrint G3 Human Gene Expression 8x60K v2 microarray chips containing 62,976 probes for 23,627 annotated genes. The hybridized chips were scanned with an Agilent Microarray Scanner D (Agilent, Santa Clara, CA). The raw data were deposited in the Gene Expression Omnibus (GEO) under accession ID GSE112530.

### Identification of DEGs

The scanned log_2_-intensities of individual samples were normalized using the quantile normalization method^57^. To identify DEGs, an integrative statistical method was applied to the normalized log_2_-intensities as previously reported^60^. Briefly, for each gene, we performed Student’s *t*-test and log_2_-median-ratio test to obtain *T* values and log_2_-median-ratios between each senescence condition and its correlated control condition (e.g., LMB-treated HDFs versus young HDFs). Then, empirical null distributions of the *T* values and log_2_-median-ratios were estimated by random sampling experiments performed 1,000 times. The adjusted *P* values of the two tests were calculated by two-tailed tests on the basis of the observed *T* values and log_2_-median-ratios. The adjusted *P* values obtained from these two tests were combined to compute an overall *P* value using Stouffer’s method^61^. The DEGs were then identified as the genes with overall *P* values < 0.05 and absolute log_2_-median-ratio > the cutoff, which was determined as the mean absolute values of the 2.5th and 97.5th percentiles in the empirical null distribution for the log_2_-median-ratio.

### DEG cluster analysis

A log_2_-fold-change (log_2_-median-ratio) matrix was formed for the 6,836 DEGs identified through the comparisons performed in this study. After quantile normalization of the log_2_-fold-changes in the matrix, we applied NMF clustering to the normalized log_2_-fold-change matrix as previously described in Kim et al.^30^. In summary, the number of clusters (*k*) was set to 100 after several trials (50, 70, 100, and 150), and adjusted *P* values were computed through the following steps: (1) The elements of the log_2_-fold-change matrix were randomly permuted; (2) NMF clustering was performed on the randomly permuted matrix with *k* = 100; (3) steps 1 and 2 were repeated 1000 times; (4) the empirical null distribution was estimated for the NMF activation values obtained from the random permutation experiments; and (5) the adjusted *P* values for the observed NMF activation values were calculated by right-sided test using the empirical distribution. The genes belonging to cluster *i* were identified as those with adjusted *P* values for the data cluster that were less than 0.01. Finally, to prioritize the major clusters according to their significance, we ordered the clusters in a descending order by the Euclidean norms of the NMF activation values, and the top 10 clusters were then selected for further analysis. Finally, we performed enrichment analysis of the GOBPs and KEGG pathways for the genes in the top 10 clusters using DAVID software^59^. GOBPs and KEGG pathways enriched by the genes in each cluster were identified as those with *P* < 0.1 and ≥5 genes.

### Evaluation of similarities in proteomes and transcriptomes between RS and NBIS models

To measure similarities of altered proteomes between RS and LMB-NBIS in the cytoplasm or nucleus, we calculated the Pearson correlation coefficient of log_2_-fold-changes (Senescent/Young or LMB/Young) of DE peptides in at least one of RS and LMB-NBIS model (Fig. 2h, i). To assess the significance of the correlation coefficients, we first selected the DE sibling peptides (*m*) with alteration directions consistent with those of the corresponding DEPs (Supplementary Methods). We then generated an empirical null distribution for the correlation coefficient by calculating correlation coefficients of the log_2_-median-ratios from random sample permutations (5000 times). Finally, the adjusted *P* values of the observed correlation coefficients were calculated by the right-sided test using the empirical distribution. This empirical testing was performed separately for the nuclear and cytoplasmic fractions. To examine the similarities of altered transcriptomes in the seven cellular senescence models, we performed hierarchical clustering on the basis of log_2_-fold-changes of the union of the DEGs identified in the seven senescence models and qualitatively evaluated the similarities between the senescence models using a dendrogram (Fig. 5a), rather than by the statistical evaluation used for the proteome.

### Cellular process network analysis

We reconstructed two process networks for GOBPs and KEGG pathways enriched by upregulated (C1-5) and downregulated clusters (C6-10). Each process network consists of GOBPs/KEGG pathways (nodes) enriched by at least one of the top 10 clusters. For each node, we obtained a list of genes annotated with the corresponding GOBP/KEGG pathway. Each pair of nodes was connected with an edge if the number of shared genes ≥ 3. Each edge connecting nodes *i* and *j* was then quantified by measuring the Sørensen–Dice coefficient, as previously described^62,63^, which is defined by 2|*P*_*i*_ ∩ *P*_*j*_ | /(|*P*_*i*_ | + |*P*_*j*_ | ), where |*P*_*i*_ | and |*P*_*j*_ | are the numbers of genes in nodes *i* and *j* and |*P*_*i*_ ∩ *P*_*j*_ | is the number of shared genes. Finally, only the edges with Sørensen–Dice coefficients > 0.4 were included in the process network, which was visualized by Cytoscape software^64^ in the organic layout option. The cutoff of 0.4 was determined on the basis of the 95^th^ percentile in the coefficient distribution of all possible pairs of nodes in the network.

### Quantitative real-time polymerase chain reaction (qRT-PCR) analysis

Total RNA was isolated from HDFs using QIAzol reagent (Qiagen) according to the manufacturer’s instructions. First-strand cDNA was synthesized using the Transcriptor First Strand cDNA synthesis kit (Roche), followed by qRT-PCR analysis (with SYBR Green I Master, Roche and a LightCycler^®^ 480 Detection System, Roche). mRNA expression levels of the target genes were calculated using the comparative threshold (CT) method and normalized to those of RPS11 (human) and CDC19 and ALG9 (replicative and chronological aging models of yeast, respectively). The primers used for PCR are listed in Supplementary Table 5.

### Comparison of senescence-associated processes between human and yeast

Gene expression profiles of young (1 generation) and old yeast cells (18-20 generations) were obtained from the GEO database (accession ID: GSE10018). After quantile normalization of the probe intensities, we compared the normalized log_2_-intensities of the young and old cells using the aforementioned integrative statistical method. In this case, to avoid bias caused by the large variation in the data, DEGs were identified as those with adjusted *P* values for the observed *T* values ≤ 0.01 and absolute log_2_-median-ratio ≥ the mean absolute values of the 5th and 95th percentiles of the empirical null distribution for the log_2_-median-ratio. We performed analysis of GOBPs and KEGG pathways for DEG enrichment using DAVID software and then compared the enriched GOBPs and KEGG pathways with those enriched by the up- and downregulated genes in the RS model.

### Yeast culture

The *Saccharomyces cerevisiae* BY4741 (MATa his3Δ1 leu2Δ0 met15Δ0 ura3Δ0) strain was used in this study. Yeast cells were cultured in YPD medium containing 1% Bacto™ yeast extract, 2% Bacto™ peptone, and 2% Difco™ dextrose (BD Bioscience) and incubated at 30 °C in an orbital shaker at 200 rpm.

### Yeast cell preparation for inducing chronological and replicative aging

For chronologically aging, yeast cells were grown in YPD medium at 30 °C in an orbital shaker at 200 rpm, and the cells were collected after 14 days (young) and 35 days (old). Replicative aging cells were prepared by rate-zonal sedimentation using a sucrose gradient. On day 3 of the stationary phase, yeast cells were sonicated and centrifuged in a linear sucrose gradient (10–30%) at 340 × *g* for 5 min. The upper band was collected as virgin cells (0 generation) and pelleted by centrifugation. The virgin cells were inoculated into fresh YPD medium and incubated at 30 °C until the optical density was doubled. Briefly, cultured cells were sonicated and centrifuged in a linear sucrose gradient (10–30%) at 340 × *g* for 5 min. The lowest band was collected as the mother cells (1 generation). The collected mother cells were incubated and separated repeatedly until the new generation was established. For preparation of total RNA, the yeast cells were resuspended in 500 μl RNA lysis buffer (10 mM EDTA, 0.5% SDS and 10 mM tris(hydroxymethyl)aminomethane buffer [pH 7.5]) and transferred to a screw cap tube containing glass beads. The cells were homogenized using a vortex at the top speed for 9 min, and the supernatant was collected by centrifugation. Total RNA was prepared using TRIzol^®^ LS reagent (Invitrogen) according to the manufacturer’s instructions with minor modifications.

### Statistical analyses

Statistical analyses for the data other than proteome and transcriptome data were performed with GraphPad Prism v.8.0 and Excel (Microsoft) software. *P* < 0.05 was considered to indicate statistically significance for all statistical analyses.

## Supplementary information

Supplementary Information
