# Supplementary material for: Disruption of nucleocytoplasmic trafficking as a cellular senescence driver
Source: Exp Mol Med. 2021 Jun 29;53(6):1092–108. doi: 10.1038/s12276-021-00643-6 (PMC8257587; doi:10.1038/s12276-021-00643-6)
Supplement: Supplementary file 1 — Supplementary Information [file 12276_2021_643_MOESM1_ESM.zip › Supplementary Information.pdf]

Supplementary Information

Disruption of nucleocytoplasmic trafficking as a cellular senescence driver.

Ji-Hwan Park[1,†], Sung Jin Ryu[2,†,‡], Byung Ju Kim[3,†, ‡], Hyun-Ji Cho[3], Chi Hyun Park[4], Hyo Jei Claudia Choi[2], Eun-Jin Jang[3], Eun Jae Yang[5], Jeong-A Hwang[5], Seung-Hwa Woo[5], Jun Hyung Lee[5], Ji Hwan Park[5], Kyung-Mi Choi[6], Young-Yon Kwon[6], Cheol-Koo Lee[6], Joon Tae Park[7], Sung Chun Cho[3], Yun-Il Lee[3], Sung Bae Lee[8], Jeong A. Han[9], Kyung A Cho[10], Min-Sik Kim[5,*], Daehee Hwang[11,*], Young-Sam Lee[3, 5,*], and Sang Chul Park[3, 12,*]

[1]Korea Bioinformation Center, Korea Research Institute of Bioscience & Biotechnology, Daejeon 34141, Republic of Korea
[2]Samsung Advanced Institute of Technology, Samsung Electronics Co. Ltd., Suwon 16677, Republic of Korea
[3]Well Aging Research Center, Division of Biotechnology, DGIST, Daegu 42988, Republic of Korea
[4]Department of Computer Science and Engineering, Kangwon National University, Chuncheon 24341, Republic of Korea
[5]Department of New Biology, DGIST, Daegu, 42988, Republic of Korea
[6]Department of Biotechnology, College of Life Sciences and Biotechnology, Korea University, Seoul 02841, Republic of Korea
[7]Division of Life Sciences, College of Life Sciences and Bioengineering, Incheon National University, Incheon 22012, Republic of Korea
[8]Department of Brain & Cognitive Science, DGIST, Daegu, 42988, Republic of Korea
[9]Department of Biochemistry and Molecular Biology, Kangwon National University School of Medicine, Chuncheon 24341, Republic of Korea
[10]Department of Biochemistry, Chonnam National University, Medical school, Gwangju 61469, Republic of Korea
[11]Department of Biological Sciences, Seoul National University, Seoul 08826, Republic of Korea
[12]The Future Life & Society Research Center, Advanced Institute of Aging Science, Chonnam National University, Gwangju 61469, Republic of Korea

[†]These authors contributed equally to this work.
[‡]Present address: UBLBio Corporation, Suwon 16679, Republic of Korea

*To whom correspondence should be addressed:
E-mail: scpark@snu.ac.kr (S.C. Park), lee.youngsam@dgist.ac.kr (Y.-S. Lee), daehee@snu.ac.kr (D. Hwang), or mkim@dgist.ac.kr (M.-S. Kim).

This file includes:

Supplementary Methods

Supplementary Figures 1-5

Legend of Supplementary Tables 1–5

– Supplementary Tables 1–5 are provided in additional Excel spreadsheets.

Supplementary References

Supplementary Methods

Peptide preparation and TMT labeling

Cells were lysed initially in Buffer A (10 mM HEPES, pH 8.0, 1.5 mM $MgCl_2$, 10 mM KCl, 300 mM sucrose, 0.1% NP-40, 0.5 mM PMSF) for 5 min on ice. The lysates were centrifuged at 10,000 x g for 1 min, and the supernatant was collected as cytosolic fraction. Then, the pellets were lysed in Buffer B (20 mM HEPES, pH 8.0, 20% glycerol, 100 mM KCl, 100 mM NaCl, 0.2 mM EDTA, 0.5 mM PMSF). After 10 min on ice, the lysates from the pellets were centrifuged at 10,000 x g for 5 min, and the supernatant was collected as nuclear fraction. After BCA measurement, a total of 200 μg of proteins from each sample were reduced with 10 mM dithiothreitol at 37° C for 45 min, alkylated with 30 mM iodoacetamide in dark at room temperature for 30 min, and diluted with 40 mM ammonium bicarbonate to reach 1 M urea. Then, the sequencing grade trypsin (Promega; Madison; WI) was used to enzymatically digest the proteome at an enzyme to protein ratio of 1 to 20 (w/w) at 37 °C overnight. After acidification, the digested peptide mixture was desalted using C18 Macro Spin Columns (HA; 74-4101). Eluted clean peptide samples were dried in a speed vacuum concentrator and kept at − 80 °C until TMT labeling. Dried desalted peptides were labeled using 10-plex TMT reagent (Thermo Scientific, Rockford, IL). TMT labeling reaction was performed as per the manufacturer instructions with minor modifications. Briefly, peptide samples were resuspended with 100 mM triethylammonium bicarbonate while TMT reagents were with 40 μL anhydrous acetonitrile. Peptide solution were mixed vigorously with the reagents and kept at room temperature for 2 hours. Finally, TMT-labeled peptides were mixed and then stored at −80 ° C until peptide fractionation.

Peptide fractionation

A complex mixture of TMT-labeled peptides were fractionated as described previously [1]. Briefly, mixed peptides were subjected to fractionation using Agilent 1260 Infinity HPLC system (Agilent, Palo Alto, CA). Accucore XL C18 analytical column (4.6 mm × 250 mm, 4 μm) was used for peptide separation. Solvent A and B were 10 mM triethylammonium bicarbonate in water (pH 7.4) and 10 mM TEAB in 90% acetonitrile (pH 7.4). Peptide fractions were collected using a 90 min linear gradient (0-5 min: 5% solvent B; 5-70 min: a shallow linear increase from 5 to 40% solvent B; 70-75 min: a sharp increase from 40 to 90% solvent B; 75-80 min: 90% solvent B; 80-85 min: 90 to 5% solvent B; 85-90 min: 5% solvent B) at the flow rate of 1 mL/min. Total number of fraction was 192 fraction which were then pooled into 24 fractions as follows: #1-#25-#49-#73-#97-#121-#145-#169, #2-#26-#50-#74-#98-#122-#146-#170, …, #24-#48-#72-#96-#120-#144-#168-#192. Peptides were vacuum-dried and stored at − 80 °C until LC-MS/MS analysis.

MS-GF+ database search and protein identification

For the individual cytoplasmic or nuclear fractions, all LC-MS/MS datasets were subjected to postexperiment-monoisotopic mass refinement (PE-MMR) analysis to accurately assign precursor masses to the MS/MS data [2]. The refined MS/MS data (i.e., mgf files) were searched against a composite database of Uniprot-Human-reference (released November, 2017; 21,200 entries) and 179 common contaminants at the target-decoy setting using MS-GF+ search engine (v10089). The MS-GF+ parameters were set as follows: i) semi-tryptic, ii) precursor mass tolerance of 10 ppm, iii) carbamidomethylation (+57.021464 Da) of cysteine and TMT labeling (+229.162932 Da) of any amino acids at N-termini and lysine as static modifications,

and iv) oxidation (+15.994915 Da) of methionine and carbamylation (+43.005814 Da) of any amino acids at N-termini as variable modifications. The search results from the 24 MS/MS datasets for cytoplasmic or nuclear fractions were combined. We then identified peptides from the peptide spectrum matches (PSMs) with false discovery rate (FDR) < 1%. Peptide abundances were quantified as maximum intensities of TMT reporter ions in the refined MS/MS spectra using MSnbase [3] package (v2.12.0) in R (v3.5.1).

Categorization of localization-dependent DEPs

Up- or down-regulation of each DEP in the nucleus or cytoplasm was determined from DE peptides for the DEP. To this end, we first categorized DE peptides into the following eight groups: up- ($G1_{pep}$) or down-regulated ($G2_{pep}$) only in the nucleus; up- ($G3_{pep}$) or down-regulated ($G4_{pep}$) only in the cytoplasm; up- ($G5_{pep}$) or down-regulated ($G6_{pep}$) in both the nucleus and cytoplasm; up-regulated in the nucleus and down-regulated in the cytoplasm ($G7_{pep}$); down-regulated in the nucleus and up-regulated in the cytoplasm ($G8_{pep}$). Due to only few DE peptides in $G7_{pep}$ and $G8_{pep}$ (≤ 2), G1–6_{pep} were used to determine the eight groups of DEPs (G1-8_{prot}). To define $G5_{prot}$, we first generated a list of potential DEPs containing the DE peptides in $G5_{pep}$ and then included each potential DEP in $G5_{prot}$ when the number of DE peptides for the DEP is more than two ($n(G5_{pep}) \geq 2$), and the half of $n(G5_{pep})$ is larger than the maximum of the numbers in the other peptide groups (G1-4_{pep} and $G6_{pep}$): $0.5 \times n(G5_{pep}) \geq \max(n(G1_{pep}), n(G2_{pep}), n(G3_{pep}), n(G4_{pep}), n(G6_{pep}))$. This procedure was also used to define $G6_{prot}$. Next, to define $G7_{prot}$, we generated a list of potential DEPs containing the DE peptides in $G1_{pep}$ and $G4_{pep}$ and included each potential DEP in $G5_{prot}$ when $\min(n(G1_{pep}), n(G4_{pep})) \geq 2$ and $0.5 \times \min(n(G1_{pep}), n(G4_{pep})) \geq \max(n(G2_{pep}), n(G3_{pep}), n(G5_{pep}), n(G6_{pep}))$. The same procedure was used to define $G8_{prot}$ using $G2_{pep}$ and $G3_{pep}$, instead of $G1_{pep}$ and $G4_{pep}$. Finally,

to define $G1_{prot}$, we generated a list of potential DEPs containing DE peptides in $G1_{pep}$ and included each potential DEP in $G1_{prot}$ when $n(G1_{pep}) \geq 2$ and $0.5 \times n(G1_{pep}) \geq \max(n(G2_{pep}), n(G3_{pep}), n(G5_{pep}), n(G6_{pep}))$. This procedure was used to define $G2_{prot}$ to $G4_{prot}$.

From these eight groups of DEPs, we determined up- or down-regulated proteins in the nucleus or cytoplasm (e.g., up-regulated proteins in the nucleus are the union of $G1_{prot}$, $G5_{prot}$, and $G7_{prot}$). The $\log_2$-fold-change of each DEP was defined as the median $\log_2$-fold-changes of its sibling DE peptides with consistent alteration directions to the DEP. The significance (*P* value) for each DEP was computed by combining those of the sibling DE peptides using Stouffer's method[4]. These $\log_2$-fold-changes and *P* values for DEPs were used for volcano plots.

Supplementary Figures

a

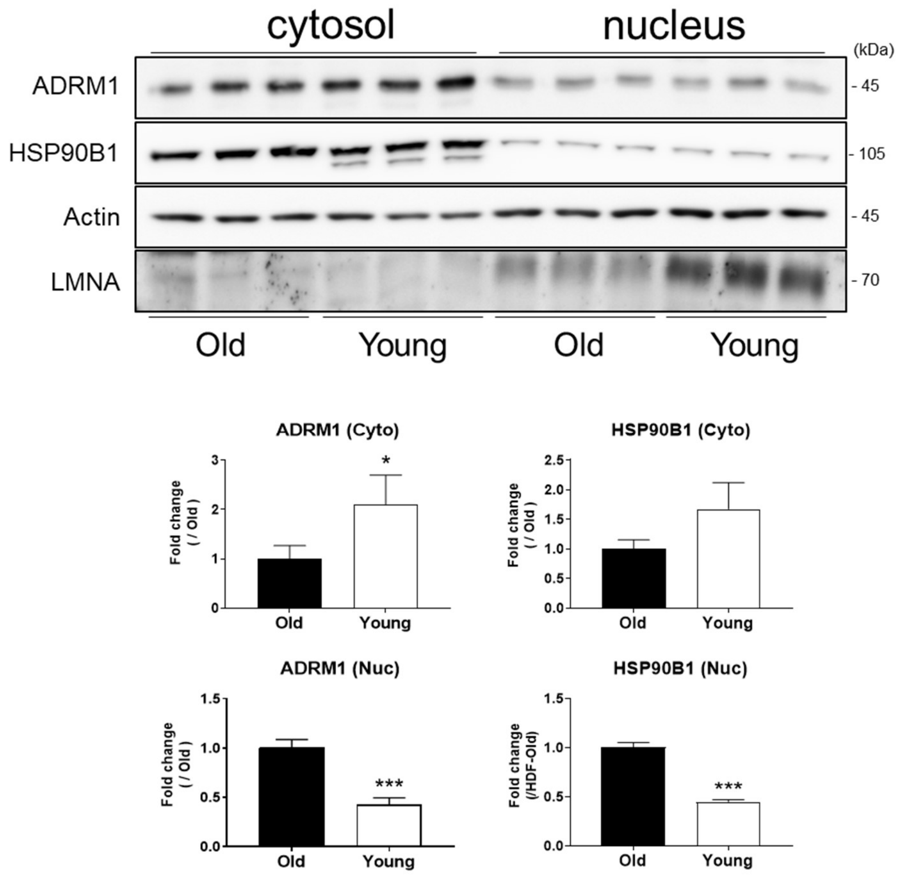

b

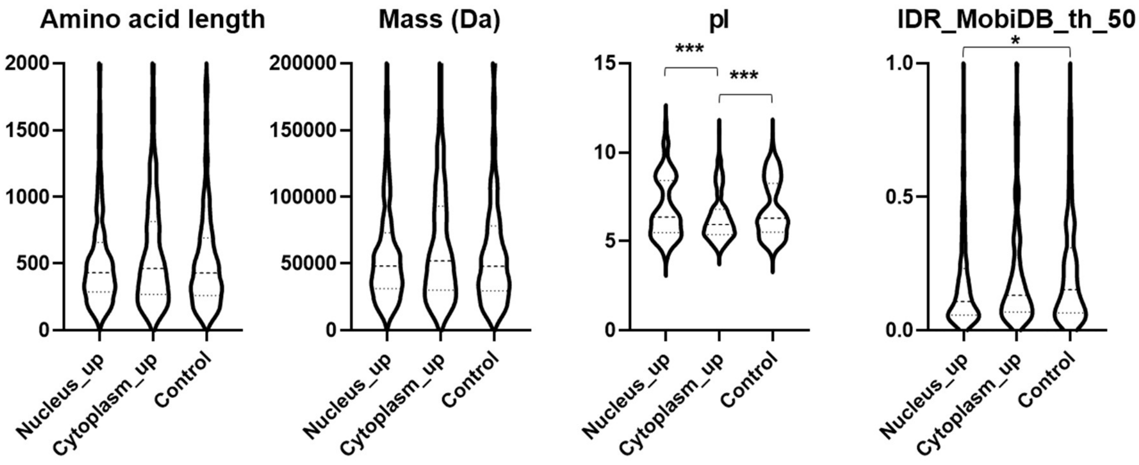

Supplementary Figure 1. **Nuclear and cytoplasmic enrichment of proteome in the presence of nuclear barrier.**

(a) Predominant accumulation of proteins in the nucleus. Immunoblot of nucleus and cytoplasm fractions from young and old using the antibodies against ADRM1 (anti-ADRM1) and HSP90B1 (anti-HSP90B1). Anti-actin, anti-LMNA and Ponceau S stained blot were used as the loading control. Values are shown as means ± SD (n = 3). *, $P < 0.05$; **, $P < 0.01$; ***, $P < 0.001$ by Student's *t*-test. **(b)** Comparison of physicochemical and structural features for proteins enriched in the nucleus and cytoplasm. The following three groups were defined for the comparison: proteins up-regulated in the nucleus (Group 1); proteins up-regulated in the cytoplasm (Group 2); and proteins with no localization changes between the nucleus and cytoplasm (Group 3). Proteins in Group 3, a control group, were selected as the proteins that had no differentially expressed peptides (P value < 0.05 and fold-change ≥ 3.53 for nucleus or 2.65 for cytoplasm) between the nucleus and cytoplasm and also more than 2 non-differentially expressed peptides (P value > 0.1 and fold-change < 1.5 for both the nucleus and cytoplasm). For each protein in the groups, we obtained amino acid length, mass, and pI from the UniProtKB/Swiss-Prot database and disordered score (IDR_MobiDB_th_50) representing the extent of disordered regions in MobiDB database. Violin plots were generated for the features of proteins in each group. *, $P < 0.05$; **, $P < 0.01$; ***, $P < 0.001$ by one-way analysis of variance (ANOVA) with Tukey's correction.

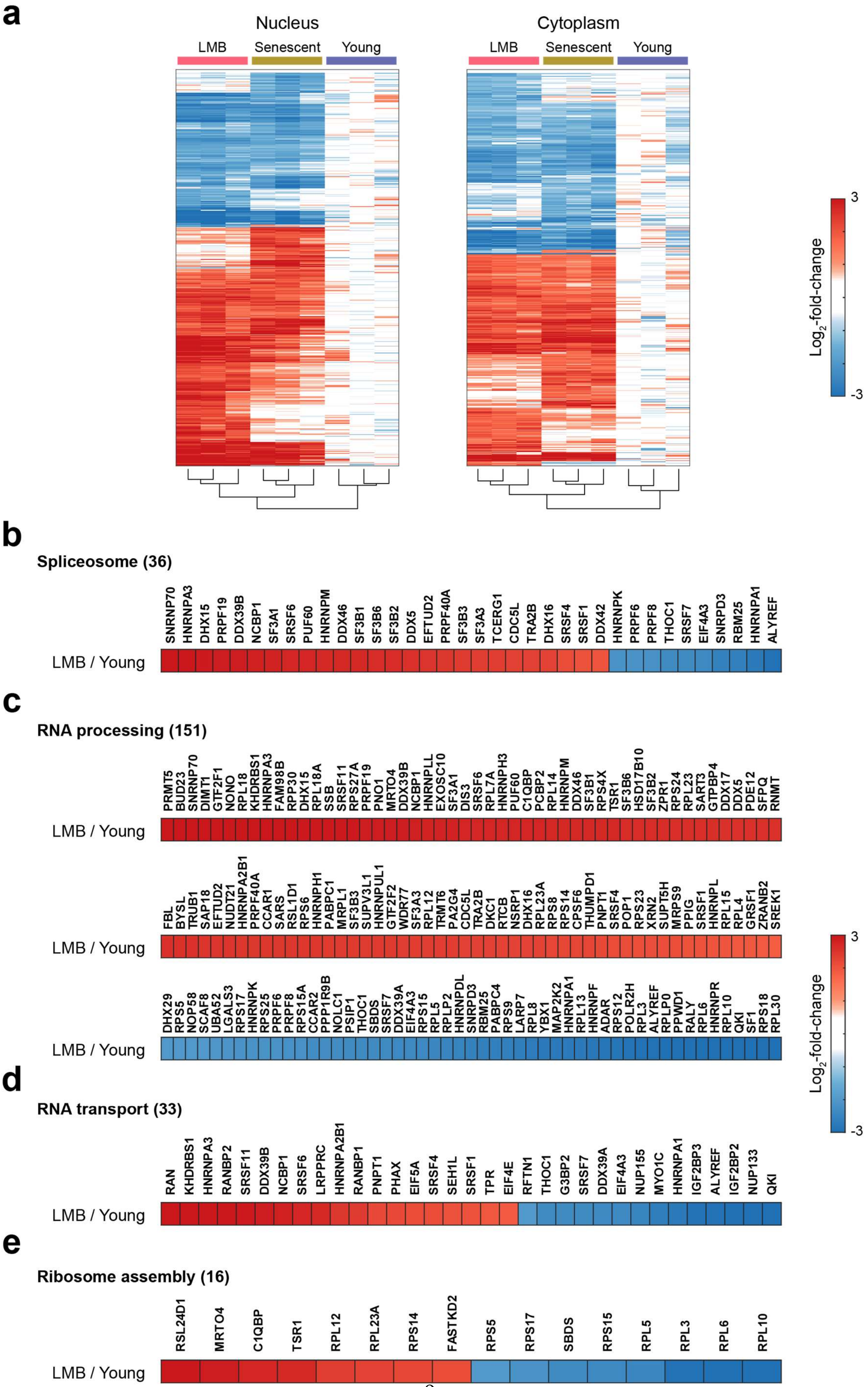

a
Nucleus
Cytoplasm
LMB
Senescent
Young
LMB
Senescent
Young
3
Log2-fold-change
-3
b
Spliceosome (36)
SNRNP70 HNRNPA3 DHX15 PRPF19 DDX39B NCBP1 SF3A1 SRSF6 PUF60 HNRNPM DDX46 SF3B1 SF3B6 SF3B2 DDX5 EFTUD2 PRPF40A SF3B3 SF3A3 TCERG1 CDC5L TRA2B DHX16 SRSF4 SRSF1 DDX42 HNRNPK PRPF6 PRPF8 THOC1 SRSF7 EIF4A3 SNRPD3 RBM25 HNRNPA1 ALYREF
LMB / Young
c
RNA processing (151)
PRMT5 BUD23 SNRNP70 DIMT1 GTF2F1 NONO RPL18 KHDRBS1 HNRNPA3 FAM98B RPP30 DHX15 RPL18A SSB SRSF11 RPS27A PRPF19 PNO1 MRTO4 DDX39B NCBP1 HNRNPLL EXOSC10 SF3A1 DIS3 SRSF6 RPL7A HNRNPH3 PUF60 C1QBP PCBP2 RPL14 HNRNPM DDX46 SF3B1 RPS4X TSR1 SF3B6 HSD17B10 SF3B2 ZPR1 RPS24 RPL23 SART3 GTPBP4 DDX17 DDX5 PDE12 SFPQ RNMT
LMB / Young
FBL BYSL TRUB1 SAP18 EFTUD2 NUDT21 HNRNPA2B1 PRPF40A CCAR1 SARS RSL1D1 RPS6 HNRNPH1 PABPC1 MRPL1 SF3B3 SUPV3L1 HNRNPUL1 GTF2F2 WDR77 SF3A3 RPL12 TRMT6 PA2G4 CDC5L TRA2B DKC1 RTCB NSRP1 DHX16 RPL23A RPS8 RPS14 CPSF6 THUMPD1 PNPT1 SRSF4 POP1 RPS23 XRN2 SUPT5H MRPS9 PPIG SRSF1 HNRNPL RPL15 RPL4 GRSF1 ZRANB2 SREK1
LMB / Young
DHX29 RPS5 NOP58 SCAF8 UBA52 LGALS3 RPS17 HNRNPK RPS25 PRPF6 PRPF8 RPS15A CCAR2 PPP1R9B NOLC1 PSIP1 THOC1 SBDS SRSF7 DDX39A EIF4A3 RPS15 RPL5 RPLP2 HNRNPDL SNRPD3 RBM25 PABPC4 RPS9 LARP7 RPL8 YBX1 MAP2K2 HNRNPA1 RPL13 HNRNPF ADAR RPS12 POLR2H RPL3 ALYREF RPLP0 PPWD1 RALY RPL6 HNRNPR RPL10 QKI SF1 RPS18 RPL30
LMB / Young
3
Log2-fold-change
-3
d
RNA transport (33)
RAN KHDRBS1 HNRNPA3 RANBP2 SRSF11 DDX39B NCBP1 SRSF6 LRPPRC HNRNPA2B1 RANBP1 PNPT1 PHAX EIF5A SRSF4 SEH1L SRSF1 TPR EIF4E RFTN1 THOC1 G3BP2 SRSF7 DDX39A EIF4A3 NUP155 MYO1C HNRNPA1 IGF2BP3 ALYREF IGF2BP2 NUP133 QKI
LMB / Young
e
Ribosome assembly (16)
RSL24D1 MRTO4 C1QBP TSR1 RPL12 RPL23A RPS14 FASTKD2 RPS5 RPS17 SBDS RPS15 RPL5 RPL3 RPL6 RPL10
LMB / Young

f

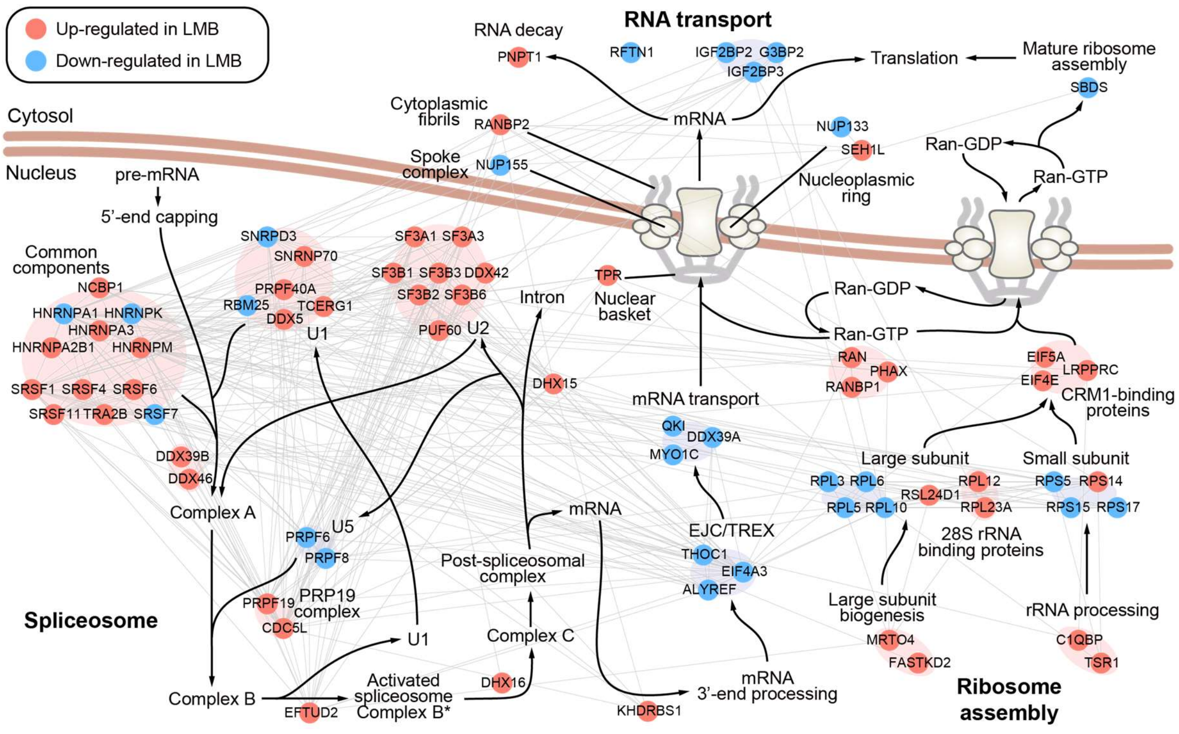

Supplementary Figure 2. **Mixed up- and down-regulation patterns of DEPs involved in spliceosome, RNA processing, RNA transport, and ribosome assembly**.

(a) Comparison of $\log_2$-fold-changes with respect to young cells between RS and LMB-treated cells. Log_2-fold-changes were calculated in individual replicates of RS and LMB-treated condition. Colors represented up- (red) and down-regulation (blue) of the differentially expressed sibling peptides with consistent alteration directions for all DEPs with respect to young cells. Hierarchical clustering (Euclidean distance as a dissimilarity measure and complete linkage) was performed to generate the dendrogram of the replicate samples. Color bar, the gradient of $\log_2$-fold-changes. (**b-e**) Heat maps showing the DEPs involved in spliceosome (**b**), RNA processing (**c**), RNA transport (**d**), and ribosome assembly (**e**). The number of DEPs belonged to each cellular process is described in parentheses. Colors represented up- (red) and down-regulation (blue) of proteins in the nucleus of LMB-treated young HDFs, compared to in the nucleus of young HDFs. Color bar, the gradient of $\log_2$-fold-

changes in LMB-treated versus young HDFs. (**f**) A network model describing interactions among the DEPs involved in spliceosome, RNA processing/transport, and ribosome assembly. Node colors represent up- (red) and down-regulation (blue) of the proteins in LMB-treated HDFs with respect to young HDFs (see legend box). Gray lines denote protein-protein interactions, arrows represent transport of molecules or biochemical reactions, and two thick lines represent the nuclear membrane. Names of complexes or functional modules for the proteins closely located are added.

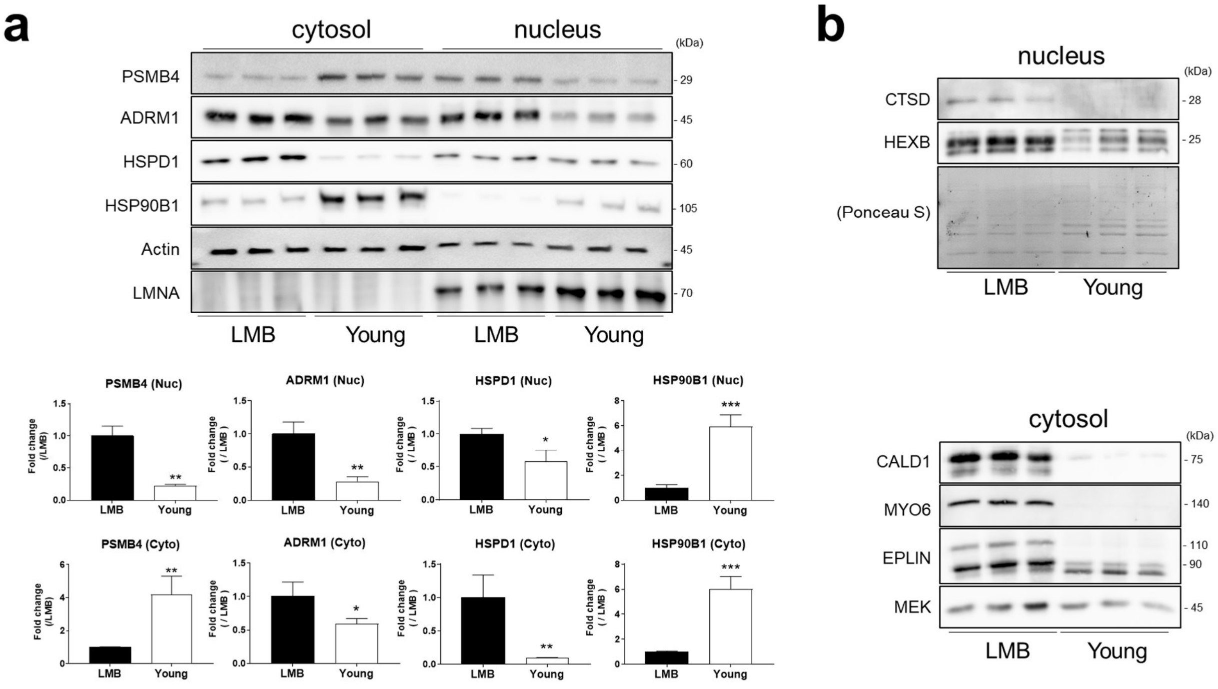

Supplementary Figure 3. **Predominant accumulation of proteins in the nucleus or cytoplasm after LMB treatment.**

(**a-b**) Immunoblot of nucleus and cytoplasm fractions from young and LMB-treated HDFs using the following antibodies: Anti-actin, anti-MEK, anti-LMNA and Ponceau S stained blot were used as the loading control. Values are shown as means ± SD (n = 3). *, $P < 0.05$; **, $P < 0.01$; ***, $P < 0.001$ by Student's *t*-test.

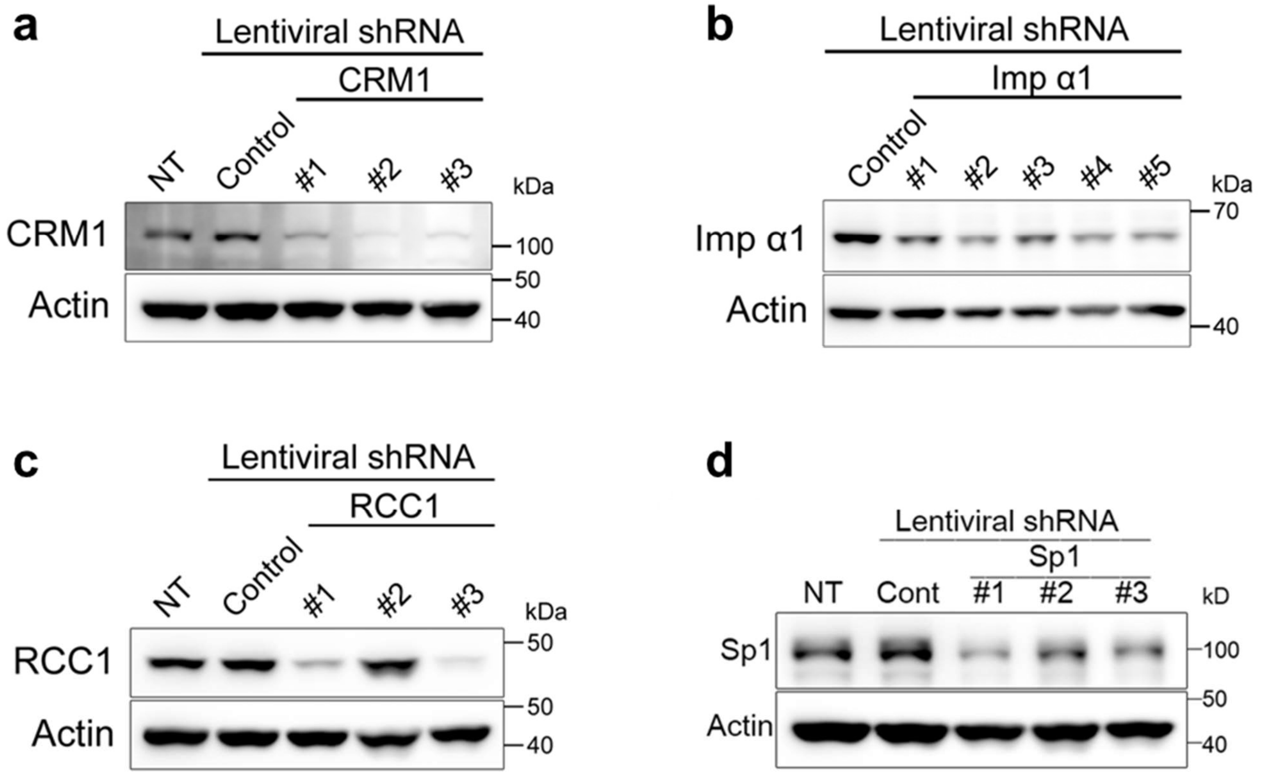

Supplementary Figure 4. Confirmation of knockdown of CRM1, importin-α1, RCC1, and Sp1 in HDFs.

(**a**-**d**) Immunoblot of total lysates from young HDFs transfected with CRM1 (**a**), importin-α1 shRNAs (**b**), RCC1 (**c**) or Sp1 (**d**). Each lane shows protein loading of cell lysates without (NT) and with transduced lentiviral control shRNA, or shRNAs against CRM1 (#1-3), against importin-α1 (#1-5), against RCC1 (#1-3) or against Sp1 (#1-3). Cells were analyzed at 72 h after infection, and anti-β actin (45 kDa) was used as the loading control.

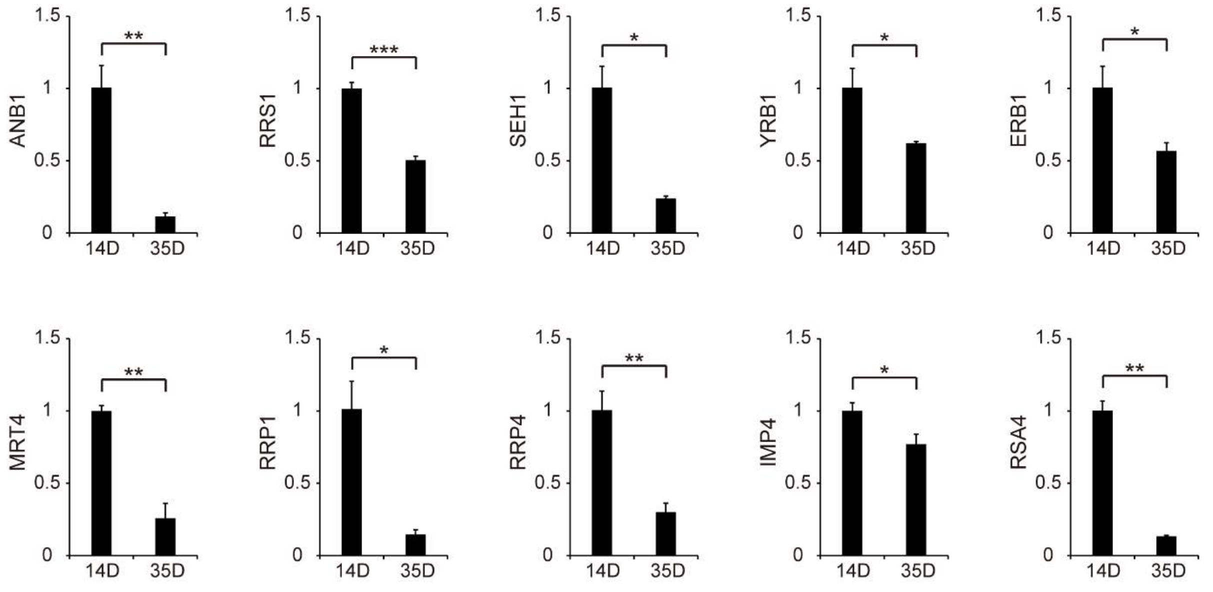

Supplementary Figure 5. qRT-PCR analysis of representative genes involved in the NBIS-RS shared cellular pathways in the yeast chronological aging model.

In the yeast chronological aging model, the analysis was done at 14 days (young) and 35 days (old). mRNA expression levels were first normalized by those of *ALG9* (internal control) and then further normalized by the mean mRNA expression level in young yeast cells. Values are means ± SD (n = 2 or 3). *, $P < 0.05$; **, $P < 0.01$; ***, $P < 0.001$ by Student's *t*-test.

Supplementary Tables

Supplementary Table 1. Detected protein groups from the nucleus and cytoplasm of young, RS, and LMB-treated HDFs.

For each protein group, Entrez ID, protein name, and description are shown together with the number of unique sibling peptides.

Supplementary Table 2. Lists of DEPs in RS and LMB-treated HDFs.

Entrez ID, protein name, and description are shown with $\log_2$-fold-changes, *P* values, and GOBPs/KEGG pathways for each DEP in the following comparisons: 1) RS HDFs versus young HDFs in the nucleus ('Nucleus_Senescent versus Young' worksheet) or the cytoplasm ('Cyto_Senescent versus Young' worksheet); and 2) LMB-treated HDFs versus young HDFs in the nucleus ('Nucleus_LMB versus Young' worksheet) or the cytoplasm ('Cyto_LMB versus Young' worksheet).

See the attached excel file named "Supplementary Table 2.xlsx".

Supplementary Table 3. DEGs between RS and young HDFs and between young and old yeast cells.

For each DEG, Entrez ID, gene name, and description are shown together with $\log_2$-fold-changes and *P* values in the comparisons: 1) RS HDFs versus young HDFs ('HDF_RS_Senescent versus Young' worksheet); and 2) old versus young yeast cells ('Yeast_Old versus Young' worksheet).

See the attached excel file named “Supplementary Table 3.xlsx”.

Supplementary Table 4. **DEGs identified in at least one of the seven senescence models, compared to their corresponding controls.**

For each DEG, Entrez ID, gene name, and description are shown together with log_2-fold-changes and *P* values in the seven comparisons: 1) RS HDFs versus young HDFs (RS/Young); 2) WGA-treated HDFs versus young HDFs (WGA/Young); 3) LMB-treated HDFs versus young HDFs (LMB/Young); 4) RCC1 shRNA-treated versus control shRNA-treated HDFs (RCC1-KD/Control); 5) HDFs overexpressing RasV12 versus control ORF (RasV12/Control ORF); 6) H_2O_2-treated HDFs versus young HDFs (H_2O_2/Young); and 7) doxorubicin-treated HDFs versus young HDFs (Doxorubicin/Young). The values were shown only for the comparisons with significant log_2-fold-changes and *P* values.

See the attached excel file named “Supplementary Table 4.xlsx”.

Supplementary Table 5. **Primers used in this study.**

See the attached excel file named “Supplementary Table 5.xlsx”.

Supplementary References

1 Kim, M. S. *et al.* A draft map of the human proteome. *Nature* **509**, 575-581, doi:10.1038/nature13302 (2014).

2 Shin, B. *et al.* Postexperiment monoisotopic mass filtering and refinement (PE-MMR) of tandem mass spectrometric data increases accuracy of peptide identification in LC/MS/MS. *Mol Cell Proteomics* **7**, 1124-1134, doi:10.1074/mcp.M700419-MCP200 (2008).

3 Gatto, L. & Lilley, K. S. MSnbase-an R/Bioconductor package for isobaric tagged mass spectrometry data visualization, processing and quantitation. *Bioinformatics* **28**, 288-289, doi:10.1093/bioinformatics/btr645 (2012).

4 Hwang, D. *et al.* A data integration methodology for systems biology. *P Natl Acad Sci USA* **102**, 17296-17301, doi:10.1073/pnas.0508647102 (2005).
